# A pervasive role for biomass burning in tropical high ozone/low water structures

**DOI:** 10.1038/ncomms10267

**Published:** 2016-01-13

**Authors:** Daniel C. Anderson, Julie M. Nicely, Ross J. Salawitch, Timothy P. Canty, Russell R. Dickerson, Thomas F. Hanisco, Glenn M. Wolfe, Eric C. Apel, Elliot Atlas, Thomas Bannan, Stephane Bauguitte, Nicola J. Blake, James F. Bresch, Teresa L. Campos, Lucy J. Carpenter, Mark D. Cohen, Mathew Evans, Rafael P. Fernandez, Brian H. Kahn, Douglas E. Kinnison, Samuel R. Hall, Neil R.P. Harris, Rebecca S. Hornbrook, Jean-Francois Lamarque, Michael Le Breton, James D. Lee, Carl Percival, Leonhard Pfister, R. Bradley Pierce, Daniel D. Riemer, Alfonso Saiz-Lopez, Barbara J.B. Stunder, Anne M. Thompson, Kirk Ullmann, Adam Vaughan, Andrew J. Weinheimer

**Affiliations:** 1 Department of Atmospheric and Oceanic Science, University of Maryland, College Park, Maryland 20742, USA; 2 Department of Chemistry and Biochemistry, University of Maryland, College Park, Maryland 20742, USA; 3 Earth System Science Interdisciplinary Center, University of Maryland, College Park, Maryland 20742, USA; 4 NASA Goddard Space Flight Center, Greenbelt, Maryland 20771, USA; 5 Joint Center for Earth Systems Technology, University of Maryland Baltimore County, Baltimore, Maryland 21250, USA; 6 Atmospheric Chemistry Observation and Modeling Laboratory, National Center for Atmospheric Research, Boulder, Colorado 80305, USA; 7 Department of Atmospheric Sciences, Rosenstiel School of Marine and Atmospheric Science, University of Miami, Miami, Florida 33149, USA; 8 Centre for Atmospheric Science, School of Earth, Atmospheric, and Environmental Science, The University of Manchester, Manchester M13 9PL, UK; 9 Facility for Airborne Atmospheric Measurements, Cranfield MK43 0JR, UK; 10 Deparment of Chemistry, University of California, Irvine, California 92697, USA; 11 Mesoscale and Microscale Meteorology Laboratory, National Center for Atmospheric Research, Boulder, Colorado 80305, USA; 12 Wolfson Atmospheric Chemistry Laboratories, Department of Chemistry, University of York, York YO10 5DD, UK; 13 NOAA Air Resources Laboratory, College Park, Maryland 20740, USA; 14 National Centre for Atmospheric Science, Department of Chemistry, University of York, York YO10 5DD, UK; 15 Department of Atmospheric Chemistry and Climate, Institute of Physical Chemistry Rocasolano, CSIC, Madrid 28006, Spain; 16 Department of Natural Science, National Research Council (CONICET), FCEN-UNCuyo, Mendoza 5501, Argentina; 17 Jet Propulsion Laboratory, California Institute of Technology, Pasadena, California 91109, USA; 18 Department of Chemistry, Cambridge University, Cambridge CB2 1EW, UK; 19 Climate and Global Dynamics Laboratory, National Center for Atmospheric Research, Boulder, Colorado 80305, USA; 20 Earth Sciences Division, NASA Ames Research Center, Moffett Field, California 94035, USA; 21 NOAA/NESDIS Center for Satellite Applications and Research, Madison, Wisconsin 53706, USA

## Abstract

Air parcels with mixing ratios of high O_3_ and low H_2_O (HOLW) are common features in the tropical western Pacific (TWP) mid-troposphere (300–700 hPa). Here, using data collected during aircraft sampling of the TWP in winter 2014, we find strong, positive correlations of O_3_ with multiple biomass burning tracers in these HOLW structures. Ozone levels in these structures are about a factor of three larger than background. Models, satellite data and aircraft observations are used to show fires in tropical Africa and Southeast Asia are the dominant source of high O_3_ and that low H_2_O results from large-scale descent within the tropical troposphere. Previous explanations that attribute HOLW structures to transport from the stratosphere or mid-latitude troposphere are inconsistent with our observations. This study suggest a larger role for biomass burning in the radiative forcing of climate in the remote TWP than is commonly appreciated.

## Abstract (web summary)

High ozone and low water structures in the tropical western Pacific are commonly attributed to transport from the stratosphere or mid-latitudes. Here, Anderson *et al*. show these structures actually result from ozone production in biomass burning plumes and large-scale descent of air within the tropics.

## Graphical abstract

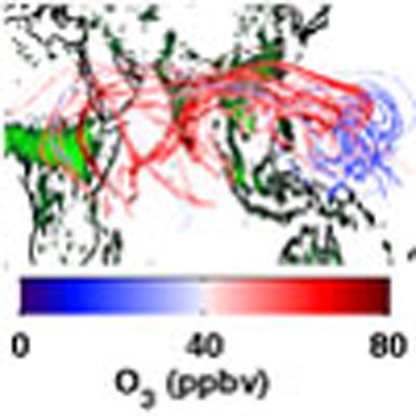

Tropospheric O_3_ is an important greenhouse gas. Ozone has exerted an increase in the global radiative forcing of climate of ∼0.4 Wm^−2^ between 1750 and 2011, almost equal to that of CH_4_ over the same time period^1^. The largest contribution to the climatic influence of O_3_ is due to enhancements over background in the tropical troposphere^2^,^3^. Elevated surface O_3_ adversely affects human health and agriculture^4^,^5^. Legislation enacted to protect public health has significantly reduced emissions of O_3_ precursors from automobiles, factories and power plants throughout the industrialized world, particularly in the Northern Hemisphere mid-latitudes^6^,^7^. Surface O_3_ levels in the industrialized extra-tropics have plateaued or fallen dramatically in response to these actions^8^,^9^. It is unclear whether the measures taken to reduce surface O_3_ in the extra-tropics will reduce the climatic impact of O_3_, since its largest radiative influence is in the tropics.

In the marine boundary layer (MBL) of the tropical western Pacific (TWP), O_3_ is removed by photochemical reactions involving halogen radicals of marine biogenic origin, resulting in O_3_ abundances of ∼20 p.p.b.v. or lower^10^,^11^. Local convection can transmit this low O_3_ air throughout the tropospheric column^12^, resulting at times in O_3_ profiles that have mixing ratios of ∼20 p.p.b.v. over an extended altitude range^13^,^14^. Air masses with elevated O_3_ are frequently accompanied by water vapour mixing ratios depressed with respect to the local background, particularly in the mid-troposphere^15^. These high ozone/low water (HOLW) structures inhibit mixing and convection^16^, alter the local radiative heating profile^17^, and affect the atmosphere's oxidative capacity^13^.

Large increases in O_3_ relative to these low background values have frequently been observed in the TWP mid-troposphere^12^,^14^,^18^,^19^,^20^,^21^,^22^,^23^,^24^,^25^. Many of these studies note that water vapour tends to be depressed, with respect to the local background, for these high O_3_ air parcels^12^,^14^,^18^,^19^,^20^,^21^,^22^,^24^, often attributing the HOLW structures to sources outside the tropical troposphere. Stoller *et al*.^19^ conclude that stratospheric air is the dominant source of HOLW air parcels observed over the TWP during multiple aircraft campaigns conducted in September and October 1991, February and March 1994 and September and October 1996. This conclusion was mainly based on the co-location of high potential vorticity (PV) with a few HOLW structures. These early campaigns lacked the instrumentation to measure the suite of chemical compounds sampled during modern campaigns, particularly the biomass burning tracer HCN. Newell *et al*.^18^ reach a similar conclusion, analysing the same data as Stoller *et al*. in addition to observations from the MOZAIC campaign from between 1994 and 1997. They note elevated CO and CH_4_ in some of their observed HOLW structures, citing entrainment of biomass burning emissions into stratospheric air. Using back trajectory analysis in addition to sonde measurements of O_3_ and H_2_O from 6 years of Southern Hemisphere ADditional OZonesonde Experiment (SHADOZ) data at three Pacific sites, Hayashi *et al*.^20^ argue that transport from the mid-latitude upper troposphere (mlUT) is the dominant source of high mid-tropospheric O_3_ in the TWP, with biomass burning only a minor contributor in some months. Kley *et al*.^12^ likewise hypothesize a mlUT origin for HOLW features near 700 hPa measured by ozonesondes in the equatorial western Pacific during the CEPEX and TOGA-COARE campaigns conducted during fall 1992. Finally, Ridder *et al*.^25^ attribute enhancements in O_3_ and CO observed remotely in the Northern Hemisphere TWP during October and November 2009 to fossil fuel combustion in Asia, Europe and North America (that is, the mid-latitudes) and in the Southern Hemisphere (SH) to both fossil fuel combustion and biomass burning.

Numerous other studies claim that low H_2_O in the TWP mid-troposphere, defined here as 300–700 hPa, is dominated by the transport of dry air from the mid-latitudes. Yoneyama and Parsons^26^ conclude horizontal advection from the mid-latitudes, likely through Rossby wave breaking, creates the observed distribution of dry air during the TOGA-COARE experiment. Combining satellite data and trajectory simulations, Waugh^27^ finds that high PV intrusions in the subtropics lead to low relative humidity (RH) in the intrusion itself and high RH ahead of the intrusion, consistent with transport from the lower stratosphere and the deep tropical troposphere, respectively. Cau *et al*.^28^ conclude that a dominant dry air source in the TWP is dehydration of air parcels via poleward movement of tropical, upper tropospheric air into the jet followed by slow descent driven by radiative cooling. This study relied on 24-day back trajectories and did not account for the presence of convective precipitation, which is known to greatly alter air mass composition. Galewsky *et al*.^29^ conclude, using a tagged tracer method and reanalysis data, that mid-latitude eddies and isentropic transport are the dominant source of low H_2_O in the subtropics in December–February 2001/2002. This analysis focused on the zonal mean distribution of H_2_O. The subtropical location of their water minimum is coincident with earth's major deserts, which means this region is decoupled from convective precipitation that rehydrates other regions of the tropics. Conversely, Dessler and Minschwaner^30^ concluded that deep convective outflow associated with the Hadley circulation is the largest source of low H_2_O in the tropical eastern Pacific. Our analysis, which focuses on the tropics (20° N–20° S) of the western Pacific, similarly suggests low water is controlled by outflow of the Hadley circulation.

Other studies cite the importance of biomass burning in controlling tropical O_3_, particularly over the Atlantic Ocean and in the SH, but fail to account for the origin of low H_2_O in HOLW air parcels. Jacob *et al*.^31^ find that tropical processes alone, including *in situ* photochemical production from biomass burning emissions and lightning NO_*x*_ (NO_*x*_=NO+NO_2_), can reproduce observed tropical O_3_ distributions using measurements over the South Atlantic during the TRACE-A campaign in September and October 1992. Other studies have attributed high O_3_ observed in the TWP to photochemical production in biomass burning plumes. Oltmans *et al*.^14^ investigated the O_3_ climatology over three locations (Fiji, Samoa and Tahiti) in the TWP from the SHADOZ network. Elevated, mid-tropospheric O_3_ was found at these sites, particularly in September and October. Back trajectory analysis connected some of these O_3_ enhancements in Fiji and Samoa to biomass burning in Indonesia, and it was noted that air parcels descended ∼3 km in transit. In the PEM-Tropics A mission conducted in the Eastern and Central Pacific of the SH in August and September 1996, both Blake *et al*.^21^ and Singh *et al*.^22^ attribute elevated O_3_ in HOLW parcels to photochemical production from biomass burning in Africa and South America based on elevated mixing ratios of biomass burning tracers (C_2_H_2_, NO_*x*_, CO, CH_3_Cl, PAN and C_2_H_6_) in the structures. Similar to Oltmans *et al*., Blake *et al*. and Singh *et al*. posit large-scale subsidence as a possible cause of the low H_2_O but offer no quantitative support for this supposition. Folkins *et al*.^24^ observed HOLW air over Fiji (18° S) at ∼120 hPa during the ASHOE/MAESA campaign in October 1994. These air parcels were enhanced in reactive nitrogen compounds and had high N_2_O mixing ratios, indicative of a tropospheric origin. They attributed the low H_2_O to transport from the mlUT using 10-day isentropic trajectories but did not provide any quantitative analysis of the effect of thermodynamics or descent on observed H_2_O. Finally, Kondo *et al*.^23^ attribute increases in O_3_ of 26 p.p.b.v. over a 31 p.p.b.v. background in the western Pacific to biomass burning during the TRACE-P campaign (February–April 2001). These studies either ignore the low H_2_O (ref. 23), offer limited explanation^14^,^21^,^22^, attribute low H_2_O to extra-tropical transport^24^, or do not describe H_2_O (ref. 31). The source of high O_3_ as well as the dynamical processes controlling the low H_2_O in HOLW structures prevalent in the TWP is clearly unresolved.

Here we analyse aircraft and ozonesonde observations, model output, and satellite data to understand the origin of the HOLW air parcels observed in the TWP during winter 2014. The Coordinated Airborne Studies in the Tropics (CAST) and CONvective TRansport of Active Species in the Tropics (CONTRAST) aircraft campaigns based in Guam (13.5° N, 144.8° E) provide a comprehensive suite of chemical measurements. We examine the 17 CAST and 11 CONTRAST flights that provided unprecedented sampling of the TWP mid-troposphere during January and February 2014 (Fig. 1). These *in situ* observations are supplemented by satellite measurements of water vapour and outgoing longwave radiation (OLR) from the daily Atmospheric Infrared Sounder (AIRS) product as well as fire counts from the Moderate Resolution Imaging Spectroradiometer (MODIS) instrument. Ten day, kinematic back trajectories were initialized along the CAST and CONTRAST flight tracks using the Hybrid Single Particle Lagrangian Integrated Trajectory (HYSPLIT) model to connect observed air parcels to their source regions. The CAM-Chem chemical transport model was run with tagged biomass burning tracers to further elucidate ozone sources and was evaluated with ozonesonde observations from the SHADOZ network. Models, satellite data and aircraft observations are used to show that anthropogenic fires in tropical Africa and Southeast Asia are the dominant source of the high O_3_ and that the low H_2_O results from large-scale descent within the tropical troposphere, after detrainment of biomass burning plumes.

## Results

### Source of high ozone in the TWP

Representative O_3_, CO and H_2_O profiles from the CAST and CONTRAST campaigns are shown in Fig. 2. Unless otherwise specified, all references to H_2_O in this paper refer to water vapour observations from an open-path laser hygrometer with complete discrimination against condensed water^32^. Mixing ratios of O_3_ and H_2_O in these profiles exhibit strong anti-correlation; that is, elevated O_3_ is closely associated with low RH^15^. These HOLW structures were a pervasive feature seen throughout both campaigns. Here we define a HOLW structure as an air parcel satisfying the simultaneous criteria of O_3_ >40 p.p.b.v. and RH <20%, where RH in this study is with respect to water for T>273 K and to ice for T<273 K. Relying solely on composition to define HOLW structures allows for the inclusion of both sharply defined, thin features (for example, Fig. 2a) as well as those that occupy a large fraction of the tropospheric column (for example, Fig. 2d). HOLW features were primarily located between 300 and 700 hPa and observed during 45 of 104 independent profiles (that is, air parcels were not intentionally resampled). For air parcels with RH >20%, median O_3_ was nearly constant with altitude at ∼20 p.p.b.v. (Fig. 3a). For air with RH <20%, median O_3_ peaked at 60 p.p.b.v. near 450 hPa, three times greater than background, and decreased to ∼40 p.p.b.v. at 750 and 300 hPa.

Kinematic trajectories connect nearly all of these HOLW structures to tropical regions with active biomass burning. Figure 4a,b shows 10-day back trajectories found using the HYSPLIT model^33^. Trajectories are coloured by observed aircraft O_3_ (invariant along each trajectory) and RH along the trajectory as output by HYSPLIT (see Methods section). Each trajectory stops at our estimate of the point of last precipitating convection, derived from a combination of cloud top height and precipitation data from geostationary satellites and the Tropical Rainfall Measuring Mission satellite. We stop the trajectories at the point of last precipitating convection because convection lofts MBL air into the upper troposphere, where it detrains throughout the column, creating a nearly uniform O_3_ profile and a water vapour profile controlled by the local saturation vapour pressure^15^. This convection effectively resets air parcel composition, removing any influence from outside the TWP. Further, global transport models are unable to reproduce the small-scale wind fields associated with deep convection, creating large uncertainty for back trajectories beyond this point. Air parcels originating from the clean SH and eastern Pacific had unimodal O_3_ and RH distributions, with medians of 22 p.p.b.v. and 63%, respectively. Trajectories originating west of the study region, primarily over Africa and Southeast Asia, exhibit a bimodal distribution^15^. Assuming a mixture of two Gaussian distributions, the two modes are described by one with peak O_3_ and RH of 21 p.p.b.v. and 60% and another with peaks of 55 p.p.b.v. and 4.5%. The low O_3_ trajectories are indicative of air parcels under local influence (that is, convectively controlled in the TWP). The high O_3_ trajectories originated over regions of active biomass burning in the tropics and reached the TWP along the upper branch of the Walker circulation (Fig. 4a). The rather substantial contribution of the HOLW structures to the overall atmospheric state of the TWP troposphere is quantified by Pan *et al*.^15^.

Simultaneous elevation of CO and O_3_ in the HOLW structures, in addition to regression analysis, suggests a tropospheric origin. Local maxima in O_3_ and CO profiles closely track one another, with *r*^2^ values between these two species ranging from 0.46 to 0.72 (Fig. 5) for the profiles shown in Fig. 2. A campaign-wide regression of CO and O_3_ for air parcels that trace back to continental regions yields *r*^2^=0.61. Photochemical enhancement ratios of O_3_ with respect to CO (ΔO_3_/ΔCO) for the profiles shown in Fig. 2 range between 0.97 and 2.85 mol mol^−1^. Mauzerall *et al*.^34^ found fresh biomass burning plumes had enhancement ratios of ∼0.15 mol mol^−1^ while plumes older than 6 days had values on the order of 0.75 mol mol^−1^. These results are consistent with Parrington *et al*.^35^ who found mean enhancement ratios of 0.81 mol mol^−1^ with a maximum ratio of 2.55 for boreal biomass burning plumes older than 5 days. Both comparisons suggest the air parcels analysed here are significantly aged and the enhancement ratios lie at the upper end of previous observations. This agrees with the back trajectories and photochemical aging analysis, discussed below, both of which yield air parcel ages of ∼10 days. NO is also elevated in these air parcels (Supplementary Fig. 1), though the correlation with O_3_ is not as prominent as for other species. This is likely due to the conversion of NO to other nitrogen-containing species, such as alkyl nitrates, peroxyacetyl nitrate and HNO_3_, none of which were measured during CAST and CONTRAST.

Regression of CO against O_3_ for all observations (Supplementary Fig. 2) reveals four distinct air mass types observed during CAST and CONTRAST: stratospheric, marine and two distinct polluted regimes, one with elevated CO, NO and O_3_ and the other with only elevated CO. Known stratospheric air, characterized by high NO and a strong anti-correlation between CO and O_3_, was encountered on two flights into the subtropics. Data collected during these two flights are excluded from the majority of the HOLW analysis, since our focus is on the tropical troposphere, but are used below to argue against a stratospheric origin of these structures. Parcels with marine characteristics (low CO, NO and O_3_) all originated from the SH or eastern Pacific (Fig. 6). Back trajectory analysis of the two polluted regimes connects the high CO, NO and O_3_ parcels to biomass burning regions in central Africa or Southeast Asia. The high CO/low NO/low O_3_ parcels have various geographic origins in Southeast Asia, which could be reflective of emissions of high CO and low NO_*x*_ from two-stroke engines, dominant in this region of the world.

Analysis of other chemical tracers suggests that the observed O_3_ in the HOLW structures is likely produced photochemically in biomass burning plumes. Both HCN and CH_3_CN are emitted almost exclusively by biomass burning^36^. Elevated mixing ratios of either species in an air parcel therefore suggests a biomass burning origin. Figure 7 shows the profiles of these two species as well as O_3_ and the industrial tracer tetrachloroethylene (C_2_Cl_4_) for the same flights as Fig. 2. Panels b–d show very tight correlations of O_3_ with HCN and CH_3_CN, while, in panel a, HCN and CH_3_CN are elevated between 400 and 700 hPa. Over the entire campaign, air parcels with back trajectories that trace back to Africa and Southeast Asia have a high correlation between O_3_ and HCN (*r*^2^=0.80), demonstrating significant biomass burning influence. The enhancement ratio of CH_3_CN to CO (4.02 p.p.t.v./p.p.b.v.) for the HOLW structures is consistent with CH_3_CN emissions from tropical forest burning (Supplementary Fig. 3)^37^ and is significantly higher than the enhancement ratio for CH_3_CN emissions from fossil fuel combustion (<0.1 p.p.t.v./p.p.b.v.)^38^. The biomass burning tracers benzene (C_6_H_6_) and ethyne (C_2_H_2_) also show strong correlation with CO (*r*^2^>0.6) as seen in Fig. 8, further confirming the origin of these air parcels.

Photochemical aging is consistent with the back trajectory analysis. Both C_6_H_6_ and C_2_H_2_ have lifetimes much shorter than that of CO (ref. 34). On the basis of modelled OH from CONTRAST, C_6_H_6_, C_2_H_2_ and CO in the TWP have lifetimes of ∼6, 12 and 35 days, respectively. All species are primarily removed by reaction with OH, so the change in CO with respect to either C_6_H_6_ or C_2_H_2_ relative to the initial emissions ratio allows determination of a photochemical age^39^. The photochemical age for C_6_H_6_ with respect to CO as determined by the Total Organic Gas Analyzer (TOGA) and Advanced Whole Air Sampler (AWAS) measurements were 13 and 8 days, respectively (see Methods section). The photochemical age for C_2_H_2_ was 11 days. These photochemical ages are broadly consistent with the elapsed time between detrainment of biomass burning plumes over Africa and Southeast Asia and transit to the TWP indicated by the back trajectory analysis (5±4 and 7±2 for Southeast Asia and Africa, respectively). The difference between the age indicated by back trajectory analysis and that of photochemical aging is likely due to dilution of biomass burning plumes with ambient air. Dilution would tend to artificially inflate the photochemical age since only CO is present in appreciable amounts in the background TWP. These relatively short values for photochemical age show the composition of air in the HOLW structures is of recent origin.

Tagged tracers for biomass burning CO in the CAM-Chem model^40^, run using assimilated meteorology, support our interpretation. A strong African biomass burning influence is frequently seen in the upper troposphere over much of the TWP and extending as far east as Hawaii, at times accounting for 17% of total CO (Supplementary Fig. 4). Deep convection can loft emissions from fires into the upper troposphere, where strong westerlies transport pollutants long distances^41^. Southeast Asian emissions are prominent throughout the tropospheric column (Supplementary Fig. 4).

### Photochemical ozone production

Analysis of ozonesonde observations, CAM-Chem O_3_, and photochemical box model output quantitatively show the high O_3_ likely originates within the tropical troposphere. Regions of tropical biomass burning have elevated O_3_ as compared with the rest of the tropics, with an O_3_ maximum over Africa and the Atlantic basin and a minimum over the TWP^42^. Median O_3_ in central Africa and Southeast Asia from CAM-Chem was ∼50 p.p.b.v. (Fig. 9a) and ∼40 p.p.b.v. (Supplementary Fig. 5a), respectively, a factor of 2 greater than background O_3_ in the TWP. We compare the CAM-Chem output to ozonesonde^43^ measurements over Nairobi (Fig. 9b) and Hanoi (Supplementary Fig. 5b), both strongly influenced by biomass burning, to evaluate CAM-Chem model performance. Mean O_3_ from the ozonesondes generally lie within 1σ of the mean CAM-Chem value, suggesting the model accurately captures the O_3_ profile in these locations. Means are used because only four or five ozonesonde profiles are available for the study period from each site. Median ozone profiles from CAM-Chem and SHADOZ show comparable agreement in the middle and upper troposphere and substantial differences near the surface (Supplementary Fig. 6). Transport of O_3_ from these biomass burning regions cannot explain all of the observed O_3_ in the HOLW structures, however, as values frequently peaked at ∼75 p.p.b.v., implying there must be photochemical production as the air parcels travel from the biomass burning region.

To estimate the net O_3_ production in the HOLW structures, a box model constrained by CONTRAST observations (see Methods section) was run for the profiles shown in Fig. 2. Net O_3_ production in the HOLW structures was on the order of ∼2 p.p.b.v. per day (Fig. 9c and Supplementary Fig. 7). The value calculated here is a lower bound to net O_3_ production in the plume. It is likely that photochemical production along the flight track is significantly lower than in fresh biomass burning plumes, which would be enriched in both NO_*x*_ and volatile organic compounds (VOCs) as compared with the more aged air observed in CONTRAST. Lightning, a significant contributor to upper-tropospheric NO_*x*_ (ref. 44), would likely further enrich fresh plumes that were lofted into the upper troposphere through deep convection. These fresh plumes can have O_3_ production rates of 7 p.p.b.v. per day or higher^42^. As the air ages, however, the abundance of HNO_3_ in the plume increases^34^, making NO_*x*_ unavailable for O_3_ production. OH will also oxidize VOCs to less reactive species, and dilution with background air will tend to counteract photochemical O_3_ production. Nevertheless, a net O_3_ production rate of ∼2 p.p.b.v. per day over 5–10 days, combined with initial O_3_ of 40–50 p.p.b.v. in the outflow of biomass burning, is consistent with the observed O_3_ in the HOLW structures.

### Low water vapour origin

We now turn to the origin of low H_2_O. As with O_3_, H_2_O observed during CAST and CONTRAST has two distinct modes, suggesting that H_2_O in the TWP is controlled both locally and by large-scale processes from outside the study region. For air parcels with HCN <150 p.p.t.v., the H_2_O profile follows the saturation vapour pressure (Fig. 3b, blue boxes), suggesting that the H_2_O mixing ratio is controlled by local thermodynamics associated with deep convection. CONTRAST flights designed to measure fresh convective outflow were the only tropical flights where HOLW structures were not observed^15^. Parcels with HCN >150 p.p.t.v. had H_2_O mixing ratios an order of magnitude lower than the local saturation vapour pressure. This is consistent with transport of dry air from outside of the TWP. Potential mechanisms to produce the dry air include horizontal advection from the mid-latitudes as well as large-scale descent in the tropics. The association of low H_2_O with high HCN indicates these air parcels originate from biomass burning regions.

AIRS measurements^45^ and trajectory analysis strongly support our supposition that the observed departures from background H_2_O result from large-scale descent in the tropics. The ascending and descending branches of the Hadley Cell, as determined by AIRS OLR, are shown in Fig. 4c as regions with OLR <250 Wm^−2^ (blue) and OLR >250 Wm^−2^ (red), respectively^46^. AIRS H_2_O averaged over the ascending branch agrees well with *in situ* H_2_O in the low HCN air parcels (Fig. 3b). Regression of *in situ* H_2_O observations from CONTRAST to co-located AIRS retrievals of H_2_O (Supplementary Fig. 8) shows these data sets are in excellent agreement (*r*^2^=0.98), allowing for direct comparison of the satellite-retrieved H_2_O to the *in situ* observations. These profiles are characteristic of deep convection, leading to RH >70%. Back trajectories show the RH <20% air parcels frequently originate in the ascending branch of the Hadley cell, flow anti-cyclonically towards the descending branch, and then reach the TWP along the prevailing westerlies (Fig. 4). Air parcels originating over Africa descend 202±64 hPa, on average, leading to significant decline in RH during transit to the TWP (Fig. 4b). Figure 3c shows the AIRS H_2_O profile over Africa (orange lines and squares). The final H_2_O profile over the TWP after subsidence (circles), assuming conservation of the H_2_O mixing ratio in transit, quantitatively agrees with *in situ* H_2_O for the enhanced HCN mode (grey symbols, Fig. 3b). Similar agreement is found for AIRS H_2_O profiles over Southeast Asia (Fig. 3d). This analysis demonstrates large-scale descent of tropical biomass burning plumes, on their transit to the TWP, can produce the dry element of the HOLW structures.

Drying of the air parcels by mixing with mid-latitude air or transport to higher latitudes is not supported by the back trajectory analysis. To determine whether air parcels were moistened or dried during transit, the difference between the trajectory starting point (that is, the trajectory initialization point along the flight track) and the H_2_O mixing ratios at the trajectory end point (that is, 10 days before observation or the point of last precipitating convection) was calculated for all HOLW structures. Water vapour mixing ratios were calculated from the RH and temperature output by the HYSPLIT model along each trajectory. Supplementary Fig. 9 shows a histogram of these changes in H_2_O. For the majority of air parcels (>80%), the H_2_O mixing ratio either increased or did not change in transit to the TWP. This moistening indicates that the majority of the HOLW structures encountered by the CAST and CONTRAST aircraft did not require additional condensation after detrainment to account for the low RH. In fact, these air parcels moistened (experienced a modest increase in H_2_O mixing ratio) in transit, likely due to mixing with background air. The low RH is due to large-scale descent in the tropics and does not require condensation in the mlUT.

### Potential origins outside the tropical troposphere

Mixing with mlUT air is inconsistent with the air mass history and observed composition of the HOLW structures. Parcel trajectories with high O_3_ began in the tropics and remained south of the jet core (Fig. 4a), indicating minimal contact with mid-latitude air. None of the back trajectories connect the HOLW air parcels to the mid-latitudes. This is consistent with the chemical composition of the filaments. Tetrachloroethylene (C_2_Cl_4_), a tracer of industrial pollution, has an atmospheric lifetime on the order of 4 months and is primarily emitted in the Northern Hemisphere mid-latitudes, creating a strong latitudinal gradient. Air masses in the tropics influenced by mid-latitude emissions from China frequently have C_2_Cl_4_ mixing ratios >2 p.p.t.v. versus a tropical background of 1 p.p.t.v. or less^47^. Tetrachloroethylene mixing ratios in the HOLW structures are a factor of 1.7 times smaller than that observed in the mid-latitude free troposphere during CONTRAST (0.89 and 1.52 p.p.t.v., respectively), suggesting little influence from the mid-latitudes. This is further confirmed by the lack of correlation between C_2_Cl_4_ and either high O_3_ (Fig. 7) or CO (Fig. 8c).

Stratospheric origin is also inconsistent with the observed composition of the HOLW structures. Elevated O_3_ in the remote TWP can have plausible sources from the polluted troposphere or the O_3_ rich stratosphere, resulting in enhancements of a similar magnitude. Mixing line analysis using measurements of at least two other tracers with distinctly different abundances in the troposphere and stratosphere can be used to assess the relative contribution of each potential source^48^. Here we use CO and H_2_O as tracers of the polluted troposphere and the stratosphere, respectively (see Methods section). The mixing line analysis shows that to reproduce the observed H_2_O profile, mixing of stratospheric and background air would require >90% stratospheric air for the majority of observed parcels (Fig. 10b) and, in turn, a stratospheric CO mixing ratio of >75 p.p.b.v. for the vast majority of the air parcels (red bars, Fig. 10c). Observed stratospheric CO was 47.6±6 p.p.b.v. (2σ; grey bar, Fig. 10c). Only ∼1% of parcels had an inferred stratospheric CO within the observed range of stratospheric CO, demonstrating a negligible role for stratospheric influence on the HOLW structures using mixing line analysis. Stratospheric influence was also estimated by interpolating PV to the calculated back trajectories. Only 4% of observed air parcels encountered stratospheric air, defined as intersecting a PV surface >2 PVU (ref. 49) along the trajectory (Fig. 10a). Relaxing the tropopause definition to 1.5 PVU does not significantly alter this percentage. The combination of low PV air along the trajectories and the mixing line analysis indicates negligible stratospheric influence on the composition of the TWP mid-troposphere during CAST and CONTRAST.

Frequent deep convection in the eastern Pacific likely prevents stratospheric air from reaching the study region. The westerly duct near 180–200° E, a region of preferential transport from the mlUT to the tropics, could potentially supply stratospheric HOLW air to the study area^50^. However, trajectories originating from the SH and eastern Pacific encountered precipitating convection ∼2.2 days before observation, that is, these trajectories do not extend back to the westerly duct. Convection promotes mixing with MBL air, removing any extra-tropical signature, as evidenced by the low O_3_ mixing ratios for air originating from the eastern Pacific as discussed above.

## Discussion

We have shown that the high O_3_ in the HOLW structures sampled in the TWP during winter 2014 is quantitatively consistent with a tropical, biomass burning source and that the low H_2_O mixing ratio is consistent with large-scale descent in the tropics. In a sense, low RH acts as a tropospheric age of air indicator in the tropics. Photochemical O_3_ production driven by emissions from biomass burning regions, in combination with large-scale descent of tropical, tropospheric air parcels that do not experience active precipitation, leads to a strong anti-correlation of H_2_O and O_3_. Prior analyses of HOLW structures, which suggested an extra-tropical tropospheric origin^12^,^18^,^19^,^20^,^25^, lacked the chemical sophistication of CAST and CONTRAST, relying primarily on ozonesondes or a limited number of chemical tracers in their analyses. Dynamical features suggested as possible mechanisms to bring dry air into the tropics—intrusions of high PV^27^, Rossby wave breaking^26^, and drying through mixing with the subtropical jet^28^,^29^—are inconsistent with the results presented here. These prior studies tend to focus on the subtropics rather than the tropics, use trajectories calculated without consideration of convective precipitation and at times in isentropic coordinates, or provide an interpretation for H_2_O that is qualitative rather than quantitative. Our aircraft and satellite data indicate, in agreement with Dessler and Minschwaner^30^, that large-scale descent in the tropics associated with the Hadley circulation exerts primary control on the H_2_O composition of the TWP troposphere for air parcels that have not experienced recent convection.

The attribution of the high O_3_ in these HOLW structures suggests a potentially larger role for biomass burning in the radiative forcing of climate in the remote TWP than is commonly appreciated. Tropical tropospheric O_3_ is a greenhouse gas, exerting a strong radiative forcing on global climate^2^,^3^. However, present efforts to limit emissions of O_3_ precursors are primarily focused on industrial activities and fossil fuel combustion that occur outside the tropics^51^. If the high O_3_ in these structures is primarily of tropical origin, then present legislation to limit the emission of O_3_ precursors in the extra-tropics may have little, if any, positive impact for the radiative forcing of climate due to tropospheric O_3_. While it is beyond the scope of this paper to estimate the radiative effects of these HOLW structures, biomass burning^52^ and HOLW^18^ structures are common features of the tropics throughout the year, implying that these structures could have a substantial impact on both global and regional radiative forcing of climate.

## Methods

### Field campaigns

**Figure 1:**
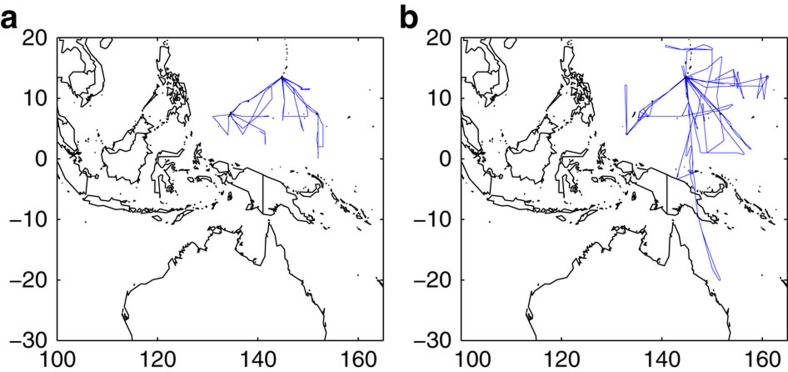
Flight tracks for the CAST and CONTRAST campaigns. Tracks for (**a**) CAST and (**b**) CONTRAST flights analysed in this study.

The CONTRAST campaign consisted of 13 research and 4 transit flights conducted using the National Center for Atmospheric Research (NCAR) Gulfstream V aircraft during January and February 2014. Objectives of the campaign included determining the budget and speciation of very short-lived halogen compounds in the TWP and investigating the transport pathways of these and other chemicals from the MBL to the tropical tropopause layer in a strongly convective environment. Research flights (RF) were either based out of Guam (13.5° N, 144.8° E) or conducted in transit between Broomfield, CO (39.95° N, 105.1° W), the home-base of the Gulfstream V, and Guam. Flights from Guam spanned latitudes from the northern coast of Australia to Japan and altitudes from ∼0.5 to 15.5 km. Transit flights (RF01, RF02, RF16 and RF17) as well as flights that sampled primarily mid-latitude air (RF06 and RF15) have been excluded from our analysis, unless otherwise indicated. Tracks of the 11 CONTRAST flights considered here are shown in Fig. 1; these flights include all that sampled exclusively in the tropics. In all, 63 vertical profiles were conducted during these flights, offering unprecedented sampling in the middle and upper troposphere of the TWP. RF15 sampled the lower, mid-latitude stratosphere near Japan, as evidenced by O_3_ mixing ratios that approached 1 p.p.m.v. The mixing ratio of stratospheric species quoted here are taken from this flight segment, where stratospheric air is defined as having O_3_ >200 p.p.b.v..

The NCAR Gulfstream V aircraft was outfitted to measure various trace gases, meteorological parameters and radiative flux. O_3_, NO and NO_2_ were measured by chemiluminescence at 1 Hz (ref. 53). The 1σ precisions of O_3_, NO and NO_2_ at 1 Hz sampling frequency, in the troposphere, are below 0.5 p.p.b.v., 10 p.p.t.v. and 20 p.p.t.v., respectively. Total uncertainties are 5% for NO and O_3_ and is 20% for NO_2_. O_3_ has been corrected for quenching owing to ambient H_2_O (ref. 54). CO was also measured at 1 Hz, with an Aero-Laser 5002 vacuum ultraviolet fluorescence instrument^55^ with a 2σ uncertainty of 3 p.p.b.v.±3%. Water vapour was measured by an open path, laser hygrometer at two wavelengths (1,853.37 and 1,854.03 nm), allowing for the sampling of H_2_O mixing ratios spanning 5 orders of magnitude^32^. Data were reported at 1 Hz with a 2σ precision of <3%. RH was calculated from observed H_2_O and temperature. Reported RH is with respect to water for temperatures above 0 °C and with respect to ice for temperatures below 0 °C. Formaldehyde (HCHO), necessary for modelling OH and HO_2_, was measured at high frequency using laser induced by the *in situ* airborne formaldehyde (ISAF) instrument^56^. HCHO was reported by ISAF at 1 Hz with a 2σ uncertainty of ±20 p.p.t.v..

TOGA measured a suite of trace gases via gas chromatography/quadrupole mass spectrometry with a sampling time of 35 s and 2 min between sampling periods^57^. Measured species relevant to this study are hydrogen cyanide (HCN), acetonitrile (CH_3_CN), tetrachloroethylene (C_2_Cl_4_), acetone (CH_3_COCH_3_), acetaldehyde (CH_3_CHO), propane (C_3_H_8_) and HCHO. AWAS also measured a suite of trace gases, including ethyne (C_2_H_2_) and benzene (C_6_H_6_). AWAS acquires up to 60 samples of ambient air per flight in electropolished stainless-steel canisters. Sampling time is pressure dependent. Canisters were analysed post-flight using gas chromatography mass spectrometry. All CONTRAST data used in this study have been averaged over the TOGA observation time, unless otherwise indicated. The sampling resolution for vertical flight segments for data averaged over the TOGA sampling period is ∼210 m.

Photolysis frequencies were calculated from up and downwelling, spectrally resolved actinic flux density by the HIAPER Airborne Radiation Package (HARP). The system uses independent, 2π steradian optical collectors connected via ultraviolet enhanced fiber optics to charged-coupled device detectors. Spectra were collected every 6 s at ∼0.8 nm resolution between 280 and 600 nm with a full-width at half maximum of 1.7 and 2.4 nm in the ultraviolet and visible, respectively. Total photolysis frequencies were calculated from the actinic flux as well as laboratory determinations of molecular cross sections and quantum yields^58^.

The CAST campaign was conducted simultaneously with CONTRAST. Whereas observations during CONTRAST were concentrated in the mid- to upper troposphere, the goal of CAST was to observe O_3_, CO, NO, very short-lived organic halogen species and radicals in the MBL and lower troposphere. Together, the two campaigns provide coverage of the TWP from just above the ocean surface to the base of the tropical tropopause layer. CAST flights were based out of Guam, Chuuk (7.4° N, 151.8° E), and Palau (7.5° N, 134.5° E) and covered altitudes from near the surface (30 m above mean sea level) to 8 km. The research portion of this campaign consisted of 23 flights during January and February 2014; 17 of these flights, as shown in Figure 1, provided observations between pressures of 300–700 hPa and are analysed here. CONTRAST and CAST flights were jointly coordinated at a shared operations center in Guam.

CAST was conducted using the Facility for Airborne Atmospheric Measurements BAe-146 aircraft. NO was measured with an air quality design, 2-channel chemiluminescence instrument through reaction with O_3_ with a 2σ uncertainty of 15%. O_3_ was measured at 0.1 Hz with a thermo environmental 49c O_3_ analyser by ultraviolet absorption with a 2σ uncertainty of ∼0.8 p.p.b.v. CO was measured with an Aero-Laser 5002 instrument at 1 Hz with a 2σ uncertainty of ∼1.4 p.p.b.v.. Water vapour mixing ratios were calculated from the observed dew point, measured with a General Eastern dew point hygrometer. HCN was measured by chemical ionization mass spectrometry^59^.

No wingtip-to-wingtip comparisons of observations for the CAST and CONTRAST campaign instruments were acquired, due to air traffic concerns in the remote TWP. To compare HCN observations, data from TOGA and the chemical ionization mass spectrometry instrument were selected for background conditions (RH >70% and O_3_ <25 p.p.b.v.) and sorted into 0.5 km bins. The mean ±1σ values of HCN for both campaigns strongly overlap, with CAST HCN slightly lower. The mean ratio of CAST to CONTRAST HCN for all altitude bins was 0.90±0.21. A similar process was used to compare O_3_ observations, selecting for measurements with RH >70%. The mean CAST to CONTRAST ratio of O_3_ values was 0.98±0.26. Since flights were conducted in different air masses and often on different days, this agreement indicates the measurements of O_3_ and HCN obtained during CAST and CONTRAST are directly comparable.

### Sondes

Profiles of ozone were measured with electrochemical concentration cell ozonesondes from SHADOZ^43^. Data used here are from observations over Hanoi, Vietnam (21.03° N, 105.85° E) and Nairobi, Kenya (1.28° S, 36.82° E) and were downloaded from the SHADOZ archive (http://croc.gsfc.nasa.gov/shadoz). Nairobi data were acquired over 5 days in January and February 2014 using a 0.5% half buffer KI solution with launch times near 8 UTC. Hanoi data were taken over 4 days in January and February 2014, using a 0.5% unbuffered KI solution and launch times near 6 UTC.

### Satellite data

Fire count data are from MODIS onboard the Terra satellite, with a local overpass time of ∼10:30 (ref. 52). The version 1 monthly product from collection 5, available at a 1 × 1° (latitude, longitude) spatial resolution, is used. MODIS data were downloaded from ftp://neespi.gsfc.nasa.gov. The Level 3, Version 6 daily AIRS product for OLR and water vapour, at 1 × 1° horizontal resolution, is also used^60^,^61^. AIRS is onboard the Aqua satellite with a local overpass time of ∼13:30. AIRS data were downloaded from ftp://acdisc.sci.gsfc.nasa.gov/ftp/data/s4pa/Aqua_AIRS_Level3. Water vapour data cited here are pressure layer averages.

### Back trajectories

Ten day kinematic back trajectories along the flight track were calculated using the NOAA HYSPLIT model^33^. RH, temperature and pressure were output along the trajectory, and H_2_O mixing ratios were calculated using the Clausius–Clapeyron relation. RH, as output by HYSPLIT, is with respect to ice for temperatures below −20 °C and a linear blend of RH with respect to ice and water for temperatures between 0 and −20 °C. HYSPLIT RH was post-processed to convert all points with temperatures between 0 and −20 °C to RH with respect to ice, to render HYSPLIT RH directly comparable to *in situ* RH.

The trajectories allowed for vertical displacement, using estimates of the vertical wind from assimilated meteorological fields. Trajectories from CONTRAST were computed along the flight track at 2 min intervals for pressures between 300 and 700 hPa, corresponding with the time between TOGA observations. CAST data were averaged over 35 s (TOGA integration time) and trajectories were calculated at 2 min intervals (TOGA sampling interval) along the flight track, to make our analysis of CAST data using trajectories analogous to the CONTRAST analysis. *Global data assimilation system* meteorological fields at 1 × 1° resolution drove the HYSPLIT model. PV, at 6 h resolution from the National Center for Environmental Prediction final analysis, was interpolated to the back trajectory through a bilinear interpolation in the horizontal and a linear interpolation in the vertical and time coordinates.

Trajectories were stopped at the point of last precipitating convection. In the TWP, convection promotes mixing with MBL air, altering air parcel composition. Precipitation rates from the tropical rainfall measuring mission satellite were combined with cloud top heights calculated from geostationary satellite infrared measurements. This technique provides coverage of the entire tropics. The convective precipitation product is available at 0.25 × 0.25° (latitude, longitude) resolution with a time step of 3 h. Intersection of a trajectory with precipitating convection was defined as a point on the trajectory being within 25 km of convection in the horizontal and being at or below the cloud top height. The 25 km radius allows for the uncertainty in the back trajectory calculation.

### CAM-Chem

The Community Atmosphere Model version 4.0 (CAM4) is the atmospheric component of the global chemistry-climate model Community Earth System Model^40^. When run with active chemistry, it is known as CAM-Chem. Here the model was run offline, with meteorological fields specified by the NASA GEOS5 model, with a horizontal resolution of 0.94° latitude × 1.25° longitude and 56 vertical levels. The model chemistry scheme includes a detailed representation of tropospheric and stratospheric chemistry (∼180 species; ∼500 chemical reactions), including very short-lived halogens. Fernandez *et al*.^62^ provide details on surface emissions, wet and dry deposition, heterogeneous reactions and photochemical processes of halogens used within CAM-Chem.

Anthropogenic emissions are from the RCP 8.5 scenario and biomass burning emissions are from the Fire INventory for NCAR (FINN)^63^. FINN combines observations of biomass burning and vegetation/land cover type from MODIS and emissions factors from multiple data sets to produce a gridded global product with a 1 × 1 km resolution. To determine the relative contributions of biomass burning from individual regions, CO emitted from fires in Africa as well as CO emitted from fires in Southeast Asia were treated as separate variables (referred to as tagged CO in the main paper).

### Box model

**Figure 2:**
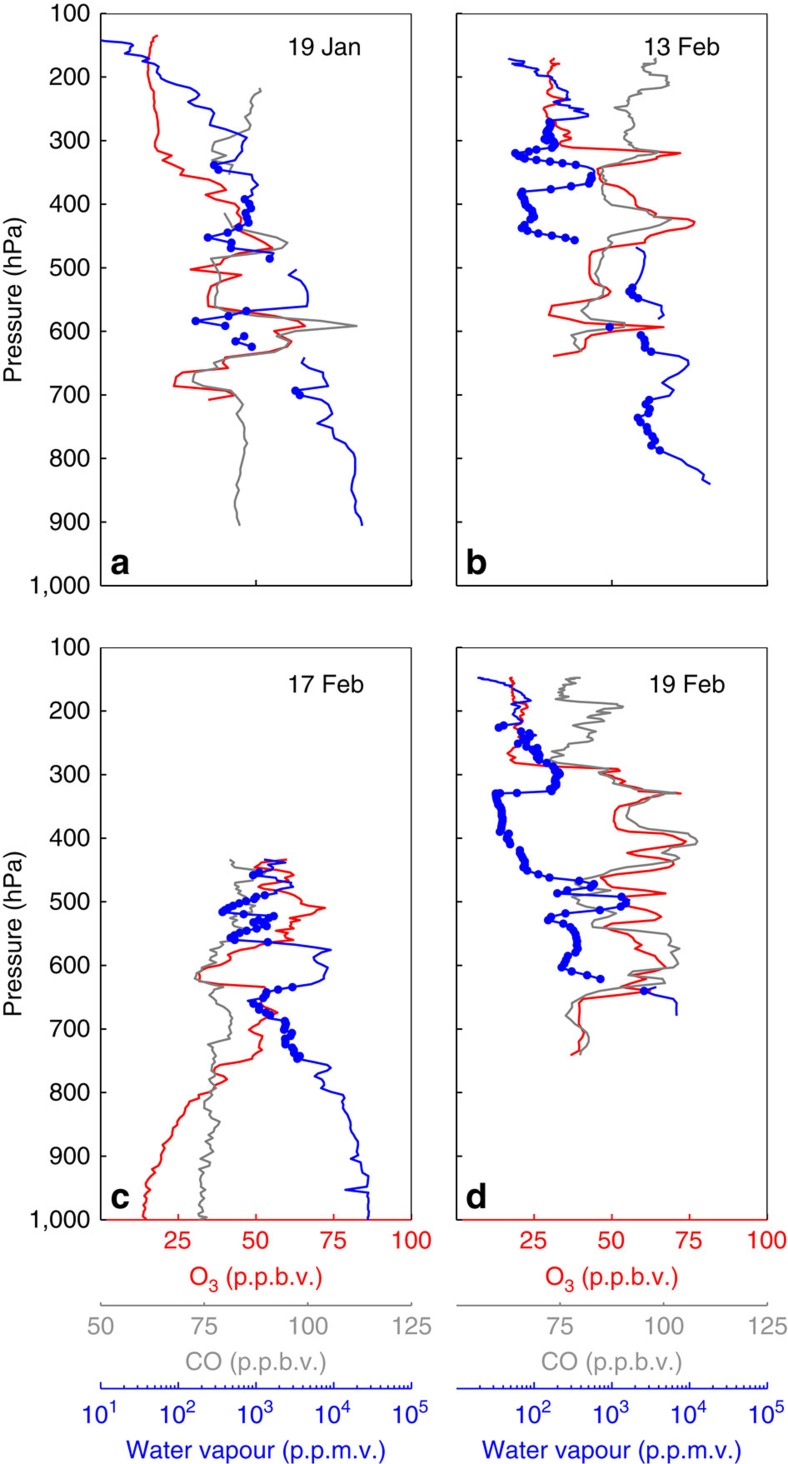
Ozone, water vapour and CO profiles in the TWP. Sample profiles of 10 s averaged O_3_ (red), CO (grey) and H_2_O (blue) from four flights during CONTRAST (**a**,**b**,**d**) and CAST (**c**). Blue circles indicate measurements of H_2_O mixing ratios for which RH <20%. RH is with respect to water and with respect to ice for temperatures above and below 273 K, respectively. Vertical profiles have a characteristic horizontal length of ∼300 km.

Net photochemical production of O_3_ was calculated using equation (1) (ref. 31) for the CONTRAST profiles shown in Fig. 2, where brackets indicate concentration and *k*_*i*_ represents the rate constant for a given reaction:


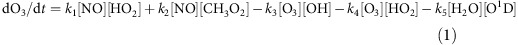


CH_3_O_2_ comprised >95% of RO_2_ for the majority of modelled points, so O_3_ production from other RO_2_ species has been ignored. HO_2_, CH_3_O_2_ and O^1^D were not measured; to determine net production of O_3_, these species were calculated using the Dynamically Simple Model of Atmospheric Chemical Complexity (DSMACC) box model^64^. Model runs were only conducted for CONTRAST flights because of lack of necessary VOC data for CAST. The model uses a subset of the Master Chemical Mechanism v3.3 (ref. 65) and was initialized with observations of methyl vinyl ketone, methacrolein, acetone, isoprene, methanol and acetaldehyde. NO_2_ was estimated using observations of j(NO_2_), O_3_, NO and modelled values of HO_2_ and CH_3_O_2_ and then used to initialize the model. The box model was constrained by Gulfstream V observations of meteorological parameters, j(NO_2_), j(O^1^D), O_3_, CO, NO, HCHO, H_2_O, C_3_H_8_ and CH_4_. The abundance of NO and j-values was allowed to vary with time of day. Photolysis frequencies for all reactions were calculated with the Tropospheric Ultraviolet and Visible Radiation model version 4.2 (ref. 66) and then scaled using observations of j(O^1^D) and j(NO_2_). Data were averaged over 10 s. Data from the TOGA instrument were linearly interpolated in time to create a 10 s data set. All output has been integrated over the diel cycle to produce 24 h mean photochemical production of O_3_, because of the diurnal variation of radical species. Model results were compared with output from another box model, the University of Washington Chemical Model (UWCM)^67^, which was constrained and initialized with the same set of input parameters. Between 300 and 700 hPa, the mean ratio of net O_3_ production found using DSMACC and UWCM was 1.01.

### Mixing line analysis

Elevated O_3_ in the remote TWP can have plausible sources from the polluted troposphere or the O_3_ rich stratosphere, imposing enhancements of a similar magnitude. Mixing line analysis using measurements of at least two other tracers with distinctly different abundances in the troposphere and stratosphere can be used to assess the relative contribution of each potential source^48^. Here we use CO and H_2_O as tracers of the polluted troposphere and the stratosphere, respectively. The fraction of stratospheric air, *f*_STRAT_ was calculated from equation (2), where H_2_O_OBS_ is the observed H_2_O mixing ratio, H_2_O_STRAT_ is the stratospheric H_2_O mixing ratio, and H_2_O_TROP_ is the altitude dependent background tropospheric H_2_O mixing ratio:


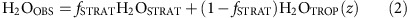


**Figure 3:**
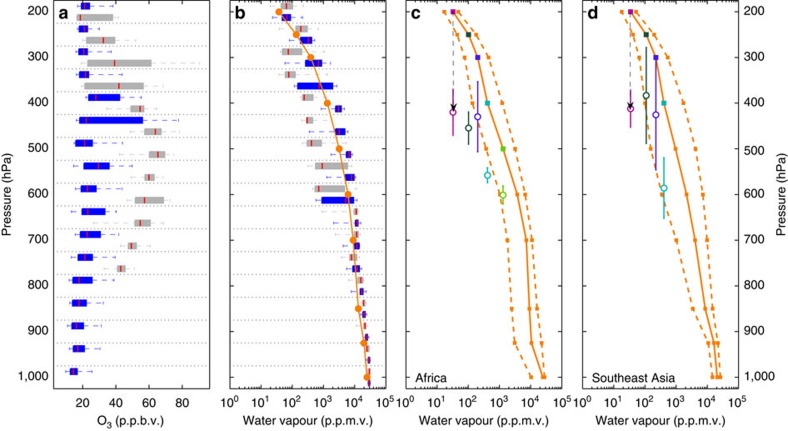
Structure of ozone and water vapour in the TWP. (**a**) Profiles of *in situ* O_3_ during CAST and CONTRAST for two modes of RH (blue, RH >20%; grey, RH <20%). Box and whisker plots show 5th, 25th, 50th, 75th and 95th percentiles for 50 hPa pressure bins. Each bin is delimited by a dotted line, and the two modes (grey and blue boxes) for a given pressure bin are offset for clarity. Between 400 and 700 hPa, the average (minimum) number of observations per blue and grey box is 650 (414) and 330 (43), respectively. Observations between 300 and 400 hPa were more limited with a minimum of 20 and a maximum of 56 observations. (**b**) Distribution of H_2_O for two modes of HCN (grey, HCN >150 p.p.t.v.; blue, HCN <150 p.p.t.v.). Median AIRS H_2_O over the ascending branch of the Hadley Cell (orange). Between 300 and 700 hPa, the average (minimum) number of observations per blue and grey box is 129 (28) and 111 (19), respectively. (**c**) Median (solid), 5th and 95th percentiles (dashed) of AIRS H_2_O over the African biomass burning region (orange). Open circles represent the mean end point of descent ±1σ of trajectories starting over the African biomass burning region and arriving over the TWP, for various initial pressures (closed squares). **d** Same as **c** but for trajectories starting over Southeast Asia.

**Figure 4:**
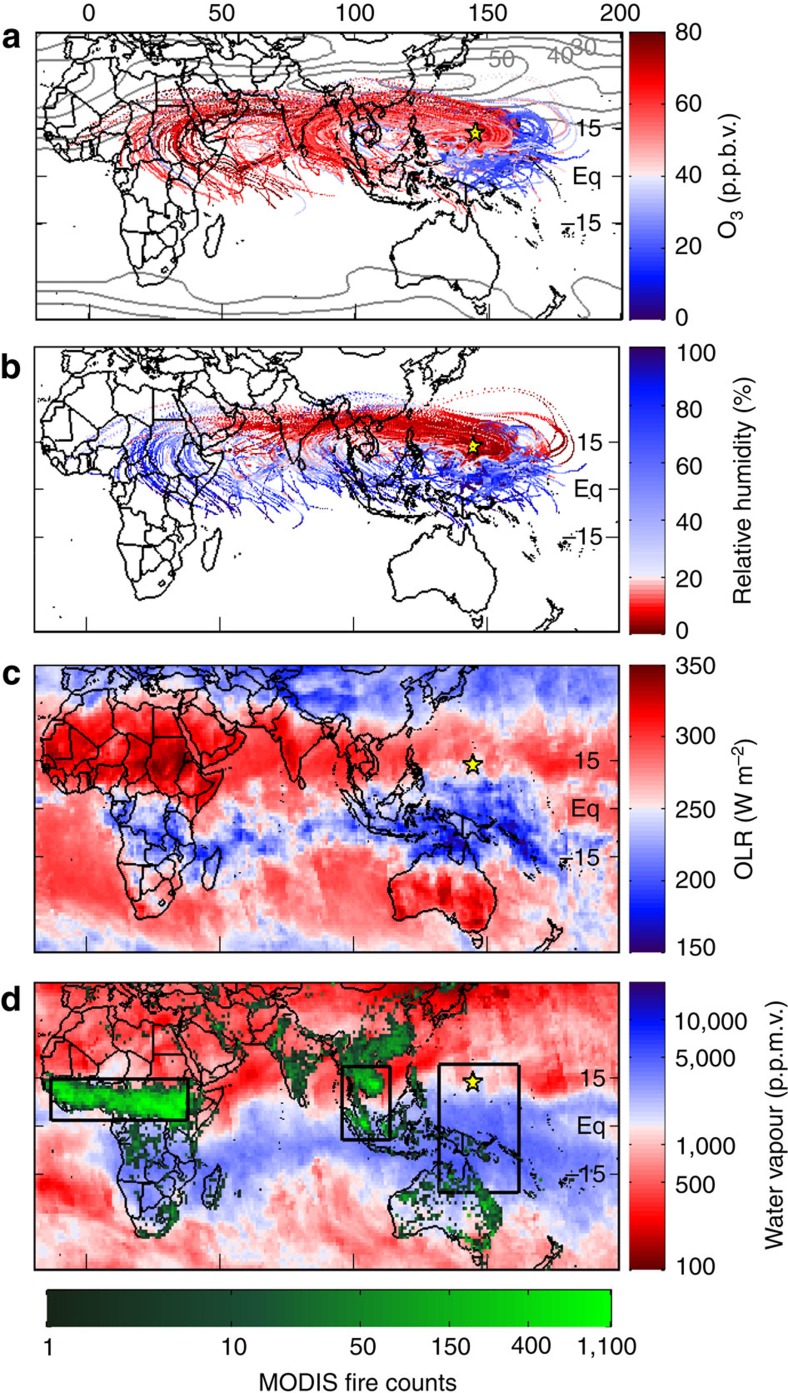
Origins of air in the TWP. (**a**) 10-day, HYSPLIT back trajectories for all CAST flights analysed here and CONTRAST RF03–05 and RF07–14 for observed pressures between 300 and 700 hPa. Trajectories are stopped when encountering convective precipitation and coloured by observed O_3_. For clarity, only every third is shown. Contours are zonal winds at 200 hPa averaged over January and February 2014 in 10 m s^−1^ intervals. The yellow star shows Guam. **b** Same as **a** but coloured by HYSPLIT RH along the trajectory (see methods). (**c**) AIRS daytime OLR averaged over CAST and CONTRAST flight days. **d** Same as **c** but for AIRS H_2_O at 500 hPa. MODIS fire counts are the total for January and February 2014. Black rectangles represent the African and Southeast Asian tropical biomass burning regions (determined subjectively by the high fire counts) and the CAST/CONTRAST study region.

**Figure 5:**
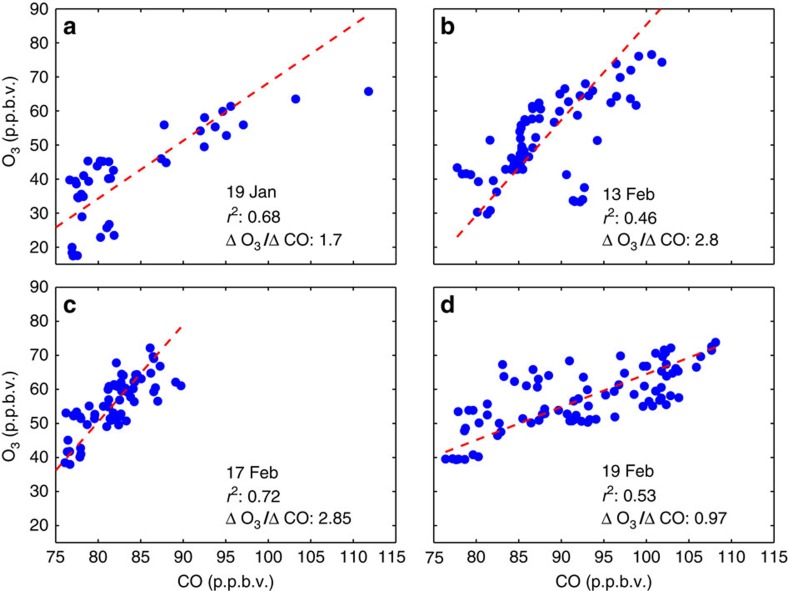
Enhancement ratios of CO and ozone from CAST and CONTRAST. Regression of CO against O_3_ for the data shown in Fig. 2 for pressures between 300 and 700 hPa (**a**–**d**). The ΔO_3_/ΔCO ratio for all profiles suggests significantly aged air, consistent with the back trajectory and photochemical aging analyses. The dashed red line is the best fit via orthogonal linear regression. Flight dates, slope and *r*^2^ values are shown for all panels.

**Figure 6:**
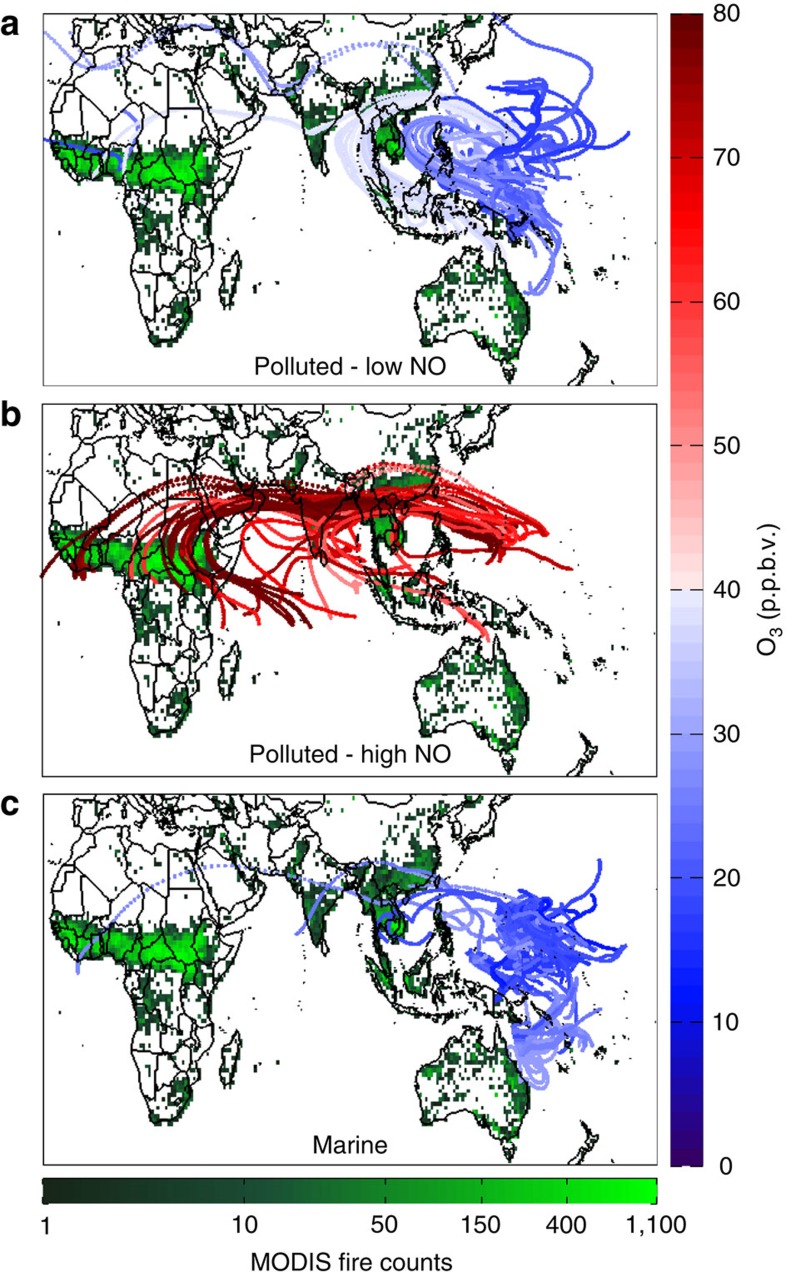
Back trajectories by chemical regime. 10-day, HYSPLIT back trajectories for all CAST flights analysed here and CONTRAST RF03–05 and RF07–14 for observed pressures between 300 and 700 hPa. Trajectories are stopped when encountering convective precipitation and coloured by observed O_3_. Trajectories are separated by the CO regimes illustrated in Supplementary Fig. 2: (**a**) Polluted—low NO (CO >95 p.p.b.v. and NO <40 p.p.t.v.), (**b**) Polluted—high NO (CO >95 p.p.b.v. and NO >50 p.p.t.v.), and (**c**) Marine (CO <80 p.p.b.v. and NO <30 p.p.t.v.). The sum of January and February 2014 MODIS fire counts is shown in green.

**Figure 7:**
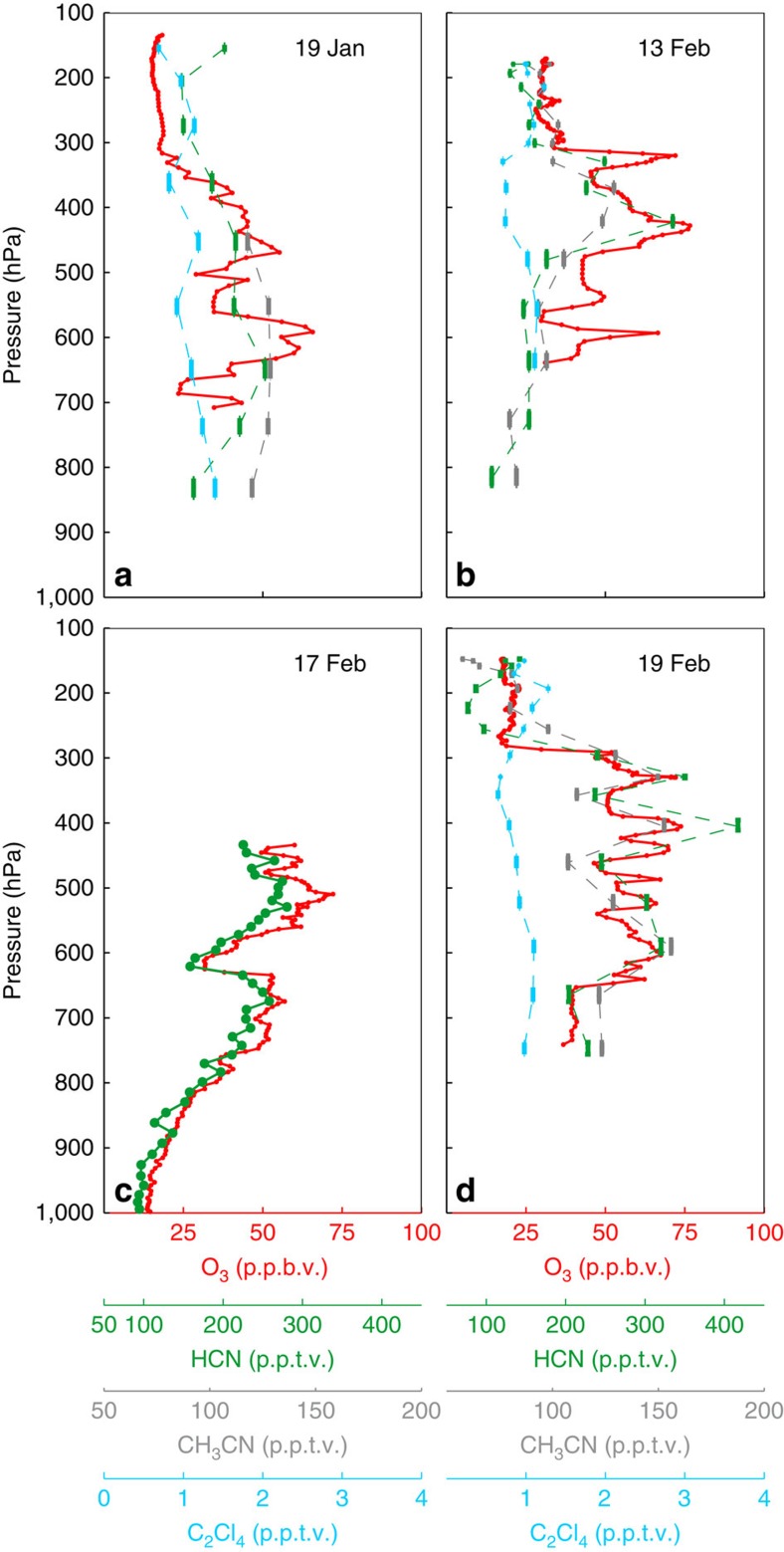
Anthropogenic tracers in the HOLW structures. Sample profiles from CONTRAST (**a**,**b**,**d**) and CAST (**c**) from the four flights shown in Fig. 2. O_3_ (red), CH_3_CN (grey), HCN (green) and C_2_Cl_4_ (light blue) are shown. O_3_ data are 10 s averages. CH_3_CN and C_2_Cl_4_ were not measured during CAST. CONTRAST HCN, CH_3_CN and C_2_Cl_4_ were sampled for 35 s at 2 min intervals. CAST HCN was sampled for 30 s. Vertical bars show the pressure range traversed during sampling.

A constant stratospheric H_2_O mixing ratio of 3 p.p.m.v. was assumed based on the mean observed H_2_O (3.1±0.1 p.p.m.v.) from RF15, which probed the lower stratosphere. Background tropospheric H_2_O was calculated by filtering 10 s averaged data for air parcels with RH >70% and O_3_ <20 p.p.b.v., leading to a profile similar to that shown by the blue bars in Fig. 3b. These data were then averaged into 1 km altitude bins.

An inferred stratospheric CO mixing ratio, denoted CO_STRAT INFERRED_ was calculated based on *f*_STRAT_ found using equation (2). The relation for CO_STRAT INFERRED_ is:


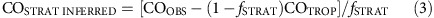


where variables have analogous definitions to those in equation (2). A value for CO_TROP_ of 85 p.p.b.v. was used, based on the median mixing ratio of CO for air parcels with RH >70%. Since *CO*_TROP_ showed little variation with altitude, a constant value was used.

### Photochemical aging

Both benzene (C_6_H_6_) and ethyne (C_2_H_2_) are tracers of biomass burning pollution and have lifetimes much shorter than that of CO (ref. 34). All species are primarily removed by reaction with OH (see reactions 1–5), so the change in CO with respect to either C_6_H_6_ or C_2_H_2_, relative to the initial emissions ratio, allows photochemical age to be determined^39^. Rate constants for reactions R3 and R4 are from IUPAC^68^ and for reactions R1, R2, and R5 from NASA JPL 2011 (ref. 69).


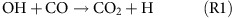



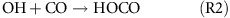



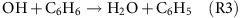



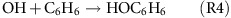



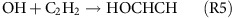


All reactions have first-order kinetics, so the ratio of the CO concentration at time *t*, [CO(*t*)], to that of benzene, [C_6_H_6_(*t*)], is described by equation (4) (ref. 39), where *k*_CO_ is the sum of the rate constants for reactions R1 and R2, *k*_C6H6_ is the sum of the rate constants for reactions R3 and R4, ER(*t*) is the enhancement ratio of CO to C_6_H_6_ at time *t*, and ER_0_ is the ratio of [CO] to [C_6_H_6_] at the time of emission. The expression for C_2_H_2_ is analogous. If all the other variables are known, the expression can be solved for *t*, the photochemical age.


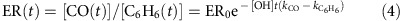


**Figure 8:**
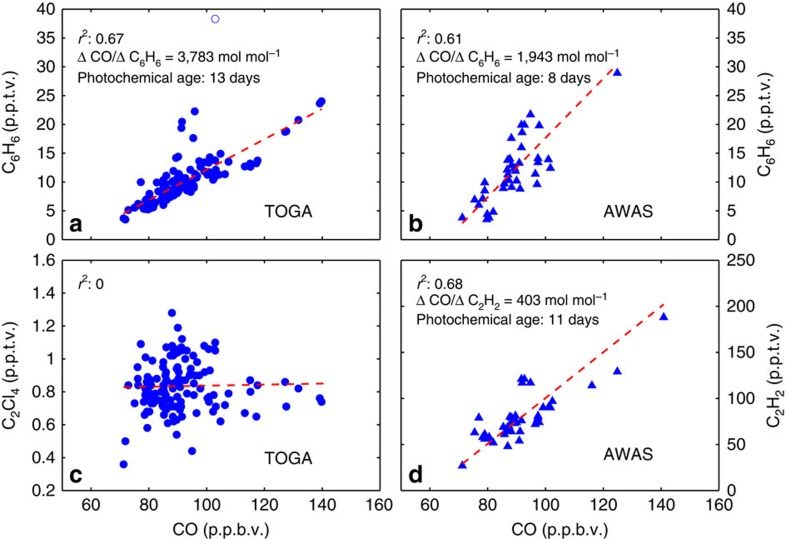
Photochemical age of observed air parcels. Regression of CO against: TOGA C_6_H_6_ (**a**); AWAS C_6_H_6_ (**b**); TOGA C_2_Cl_4_ (**c**); and AWAS C_2_H_2_ (**d**). Data are only for the HOLW structures (O_3_ >40 p.p.b.v. and RH <20%). The dashed red line is the best fit via orthogonal linear regression. A single data outlier (open circle, **a**) has been excluded from the analysis. Values of *r*^2^ are shown for all panels; enhancement ratios and photochemical ages are shown for panels **a**, **b** and **d**. Excluding the data point for which C_6_H_6_ >25 p.p.t.v. for panel **b** results in an *r*^2^ of 0.47, a CO to C_6_H_6_ enhancement ratio of 1,823 mol mol^−1^, and a photochemical age of 7 days.

**Figure 9:**
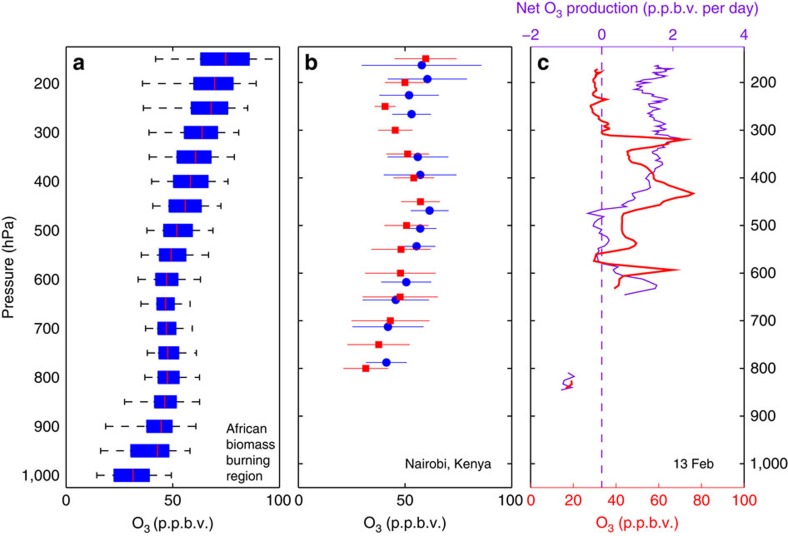
Ozone over Africa and *in situ* potochemical ozone production in the TWP. (**a**) Vertical distribution of CAM-Chem O_3_ in the African biomass burning region (that is, black box Fig. 4); 5th, 25th, median, 75th and 95th percentiles are shown. (**b**) Mean ±1σ of SHADOZ ozonesonde observations over Nairobi, Kenya for January and February 2014 (red). Mean ±1σ CAM-Chem O_3_ modelled over Nairobi sampled on the same days as the ozonesondes (blue). (**c**) O_3_ profile from Fig. 2b (red) and net O_3_ production in the profile (purple, top axis), calculated using the DSMACC photochemical box model (see Methods section).

**Figure 10:**
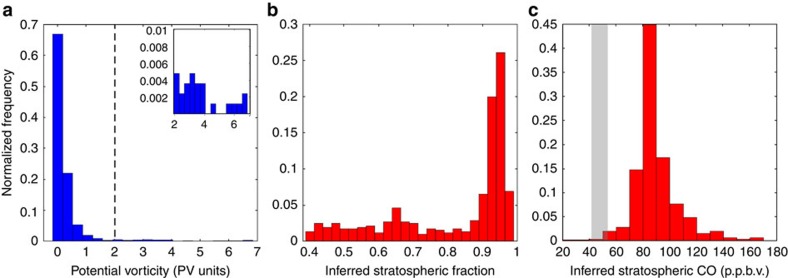
Negligible stratospheric influence on the composition of the TWP troposphere. (**a**) Distribution of the maximum absolute value of PV along the back trajectories for all air parcels observed between 300 and 700 hPa that have travelled over the southeast Asian and/or African biomass burning regions. (**b**) Distribution of the inferred stratospheric fraction of air (_STRAT_) needed to explain the low H_2_O in the HOLW filaments, if the depression in H_2_O were solely due to stratospheric intrusion. (**c**) Distribution of inferred stratospheric mixing ratio of CO (CO_STRAT INFERRED_) assuming the fraction of stratospheric air shown in **b**. Grey area represents the mean ±2σ of CO observed in the stratosphere during CONTRAST RF15 (O_3_ >200 p.p.b.v.). This mixing line analysis suggests negligible stratospheric influence on the composition of the TWP mid-troposphere during CAST and CONTRAST.

The enhancement ratios of CO to C_6_H_6_ and CO to C_2_H_2_ over the entire campaign were determined by using an orthogonal linear regression for parcels where O_3_ >40 p.p.b.v. and RH <20% (Fig. 8). The slope of these lines is the enhancement ratio. This was done over a campaign-wide basis and not for each profile because of the limited sampling frequency of C_6_H_6_ and C_2_H_2_. Separate regressions were done for the TOGA and AWAS C_6_H_6_ measurements. Values of *r*^2^ for all regressions were >0.61. The value of ER_0_ for the two relations was assumed to be 724 mol CO per mol C_6_H_6_ and 241 mol CO per mol C_2_H_2_, which are characteristic of a tropical forest^70^. The photochemical age varied by <0.5 days when emission ratios for other vegetation types^70^ were considered. Constant values of temperature (247 K), pressure (350 hPa) and [OH] (1.7 × 10^6^ cm^−3^) were assumed when calculating the photochemical age. Temperature and pressure values were the average along the back trajectory for the HOLW filaments, and 1.7 × 10^6^ cm^−3^ is the 24-h mean OH concentration for parcels between 300 and 700 hPa from our box model runs. Equation (4) also assumes that any mixing with ambient air dilutes both species equally.

### Code availability

The CAM-Chem code is available for download at www2.cesm.ucar.edu. An online version of the HYSPLIT model is available at http://ready.arl.noaa.gov/HYSPLIT.php. The DSMACC photochemical box model can be downloaded at http://wiki.seas.harvard.edu/geos-chem/index.php/DSMACC_chemical_box_model and the UWCM box model can be downloaded at https://sites.google.com/site/wolfegm/models.

## Additional information

**How to cite this article:** Anderson, D. C. *et al*. A pervasive role for biomass burning in tropical high ozone/low water structures. *Nat. Commun.* 7:10267 doi: 10.1038/ncomms10267 (2016).

## Supplementary Material

Supplementary Information Supplementary Figures 1-9
