# Supplementary material for: A pervasive role for biomass burning in tropical high ozone/low water structures
Source: Nat Commun. 2016 Jan 13;7:10267. doi: 10.1038/ncomms10267 (PMC4735513; doi:10.1038/ncomms10267)
Supplement: Supplementary Information — Supplementary Figures 1-9 [file ncomms10267-s1.pdf]

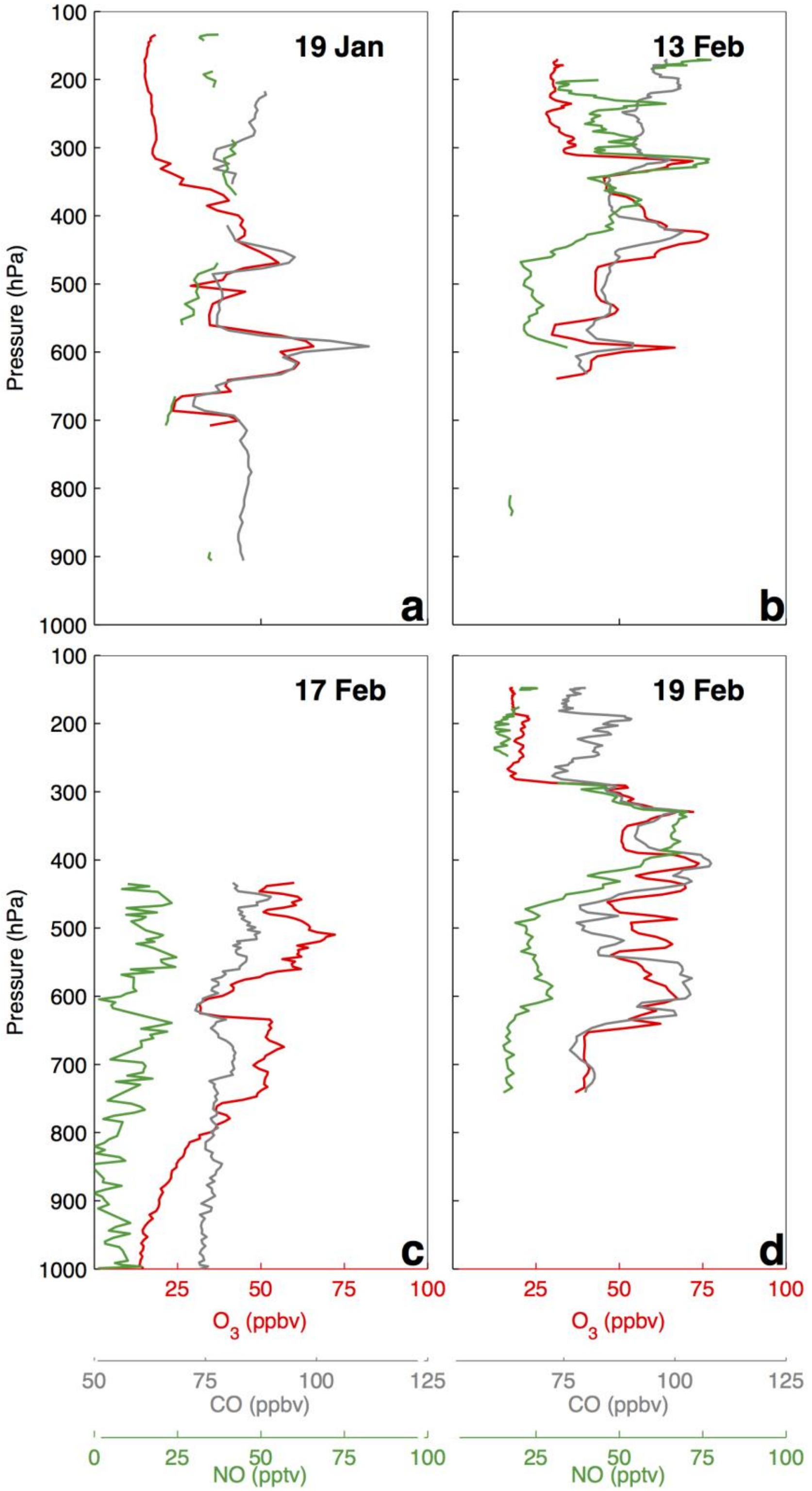

Supplementary Figure 1 | NO, CO, and ozone profiles in the TWP. Sample profiles of 10 sec averaged O_3 (red), CO (grey), and NO (green) from four flights during CONTRAST (panels **a**, **b**, and **d**) and CAST (panel **c**). These are the same profiles as shown in Figure 2. Vertical profiles have a characteristic horizontal length of approximately 300 km.

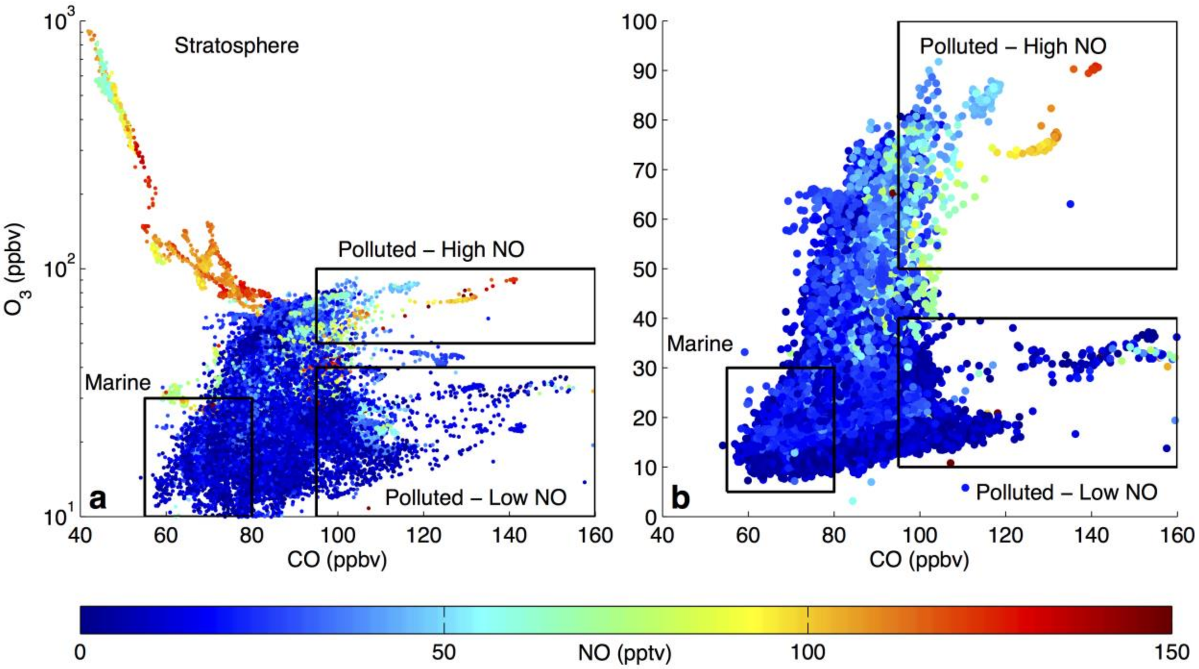

Supplementary Figure 2 | Chemical regimes of the TWP determined by CO and NO composition. 10 sec averaged O_3 regressed against CO observations, colored by NO. (**a**) Data from all CAST and CONTRAST flights. (**b**) Data analyzed in the trajectory analysis. CAST data are for the 17 flights that provided observations between 300 and 700 hPa and CONTRAST data are for RF03-05 and RF07-14.

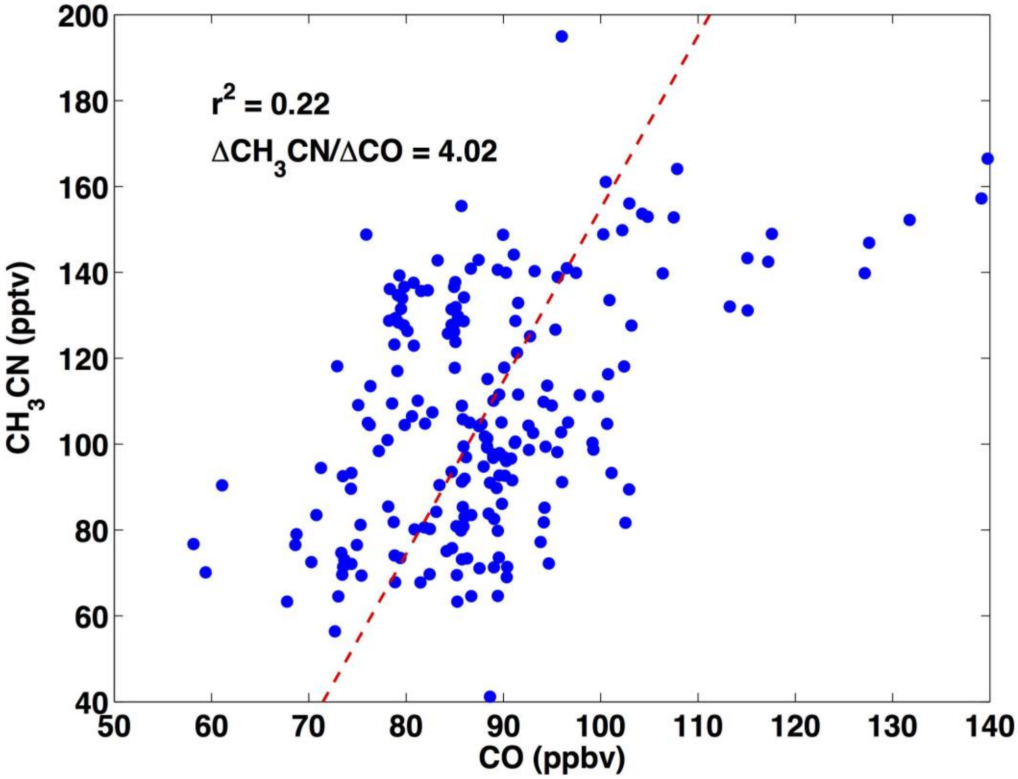

Supplementary Figure 3 | Correlation of CO with the biomass burning tracer CH_3CN. Regression of CO against acetonitrile (CH_3CN) for all observations where O_3>40 ppbv and RH<20%. The dashed red line is the best fit via orthogonal linear regression. The $\Delta CH_3CN/\Delta CO$ enhancement ratio and r^2 values are also shown. The $\Delta CH_3CN/\Delta CO$ ratio is consistent with emissions from burning of a tropical forest. Using a linear, least squares regression the $\Delta CH_3CN/\Delta CO$ enhancement ratio is 1.1 pptv/ppbv.

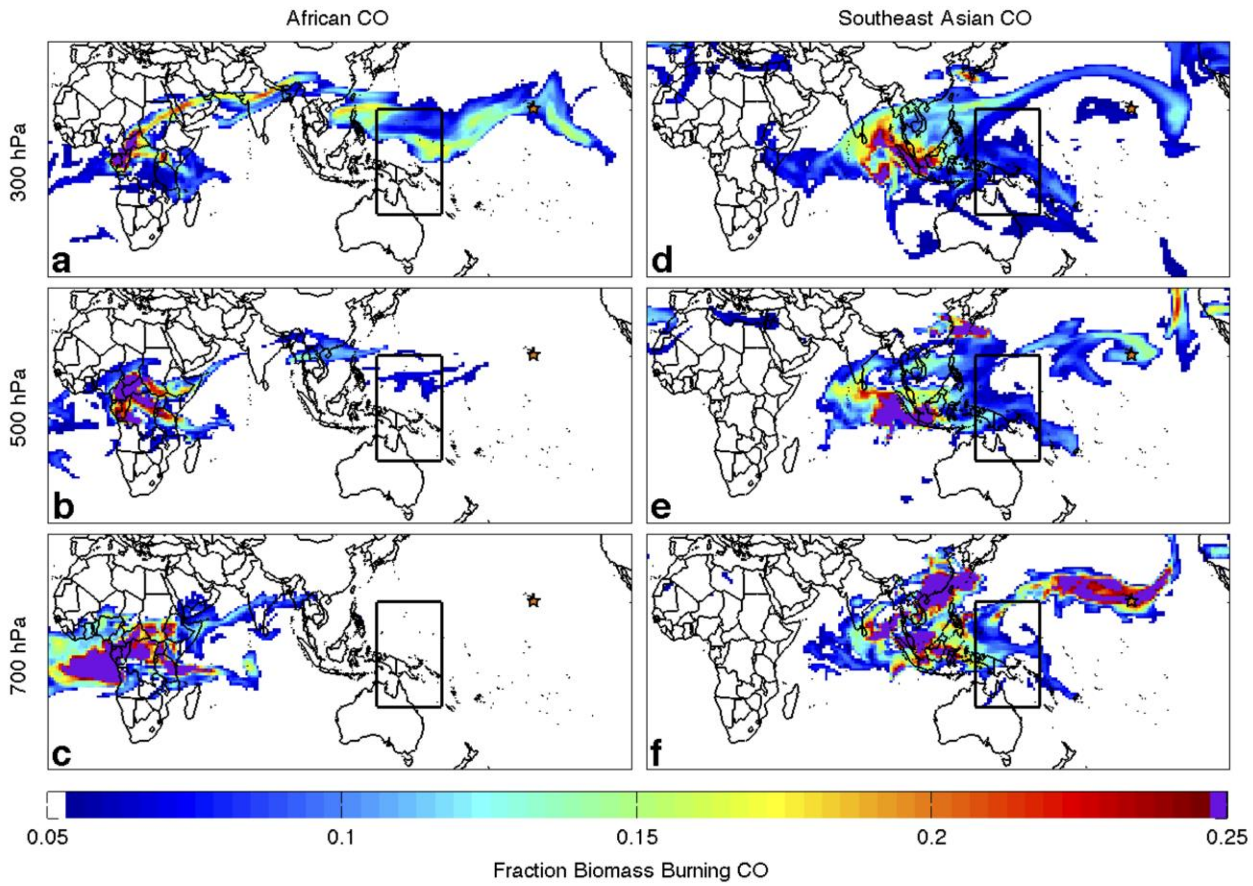

Supplementary Figure 4 | Fraction of modeled CO attributed to African and Southeast Asian biomass burning. Fraction of CO produced from biomass burning on 11 Feb 2014 from Africa (**a-c**) and 25 Feb 2014 from Southeast Asia (**d-f**) at 300 (**a** & **d**), 500 (**b** & **e**), and 700 (**c** & **f**) hPa found using tagged CO in CAM-Chem. The CAST/CONTRAST study region is shown by the rectangle and Hawaii is shown by the orange star to provide perspective in interpretation of these model results.

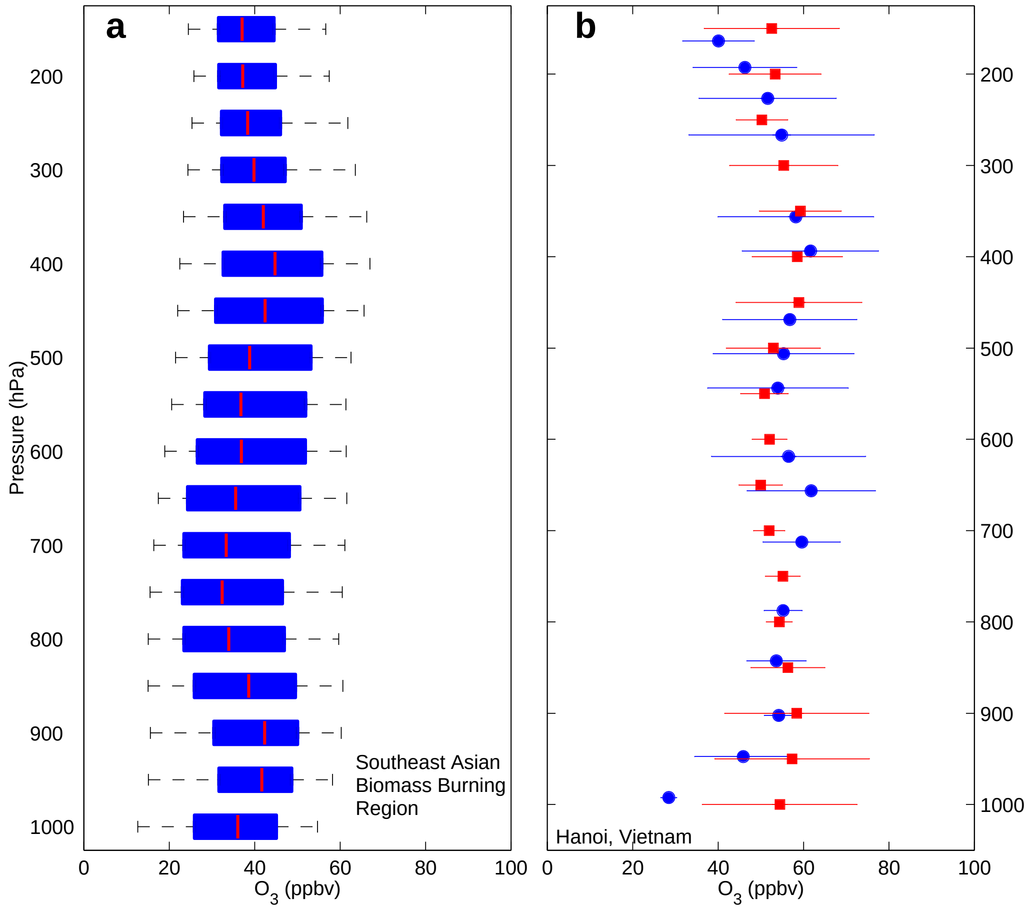

Supplementary Figure 5 | Measured and modeled ozone over Southeast Asia. (**a**) Vertical distribution of CAM-Chem O_3 in the Southeast Asian biomass burning region (i.e. black box Fig. 4); 5th, 25th, median, 75th, and 95th percentiles are shown. (**b**) Mean ±1σ of SHADOZ ozonesonde observations over Hanoi, Vietnam for January and February 2014 (red). Mean ±1σ CAM-Chem O_3 modeled over Hanoi, sampled on the same days as the ozonesondes (blue).

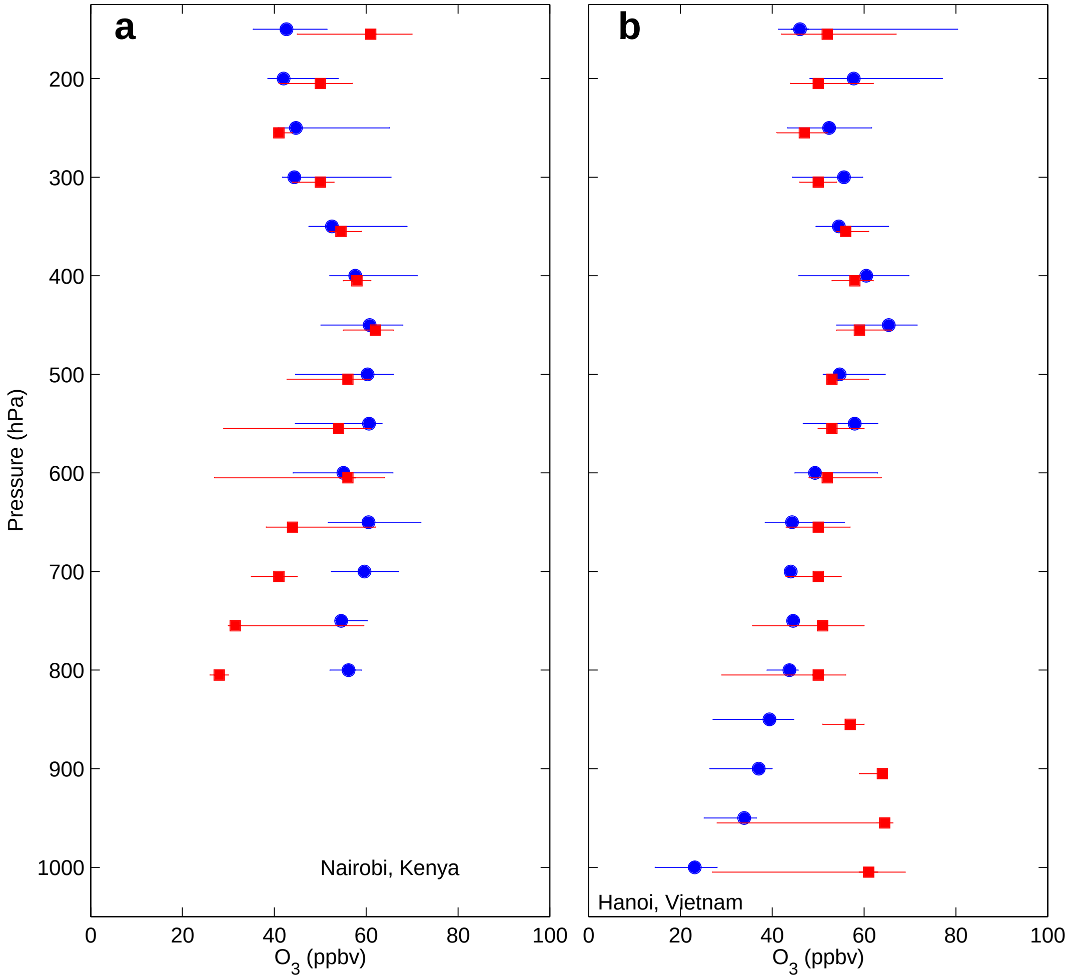

Supplementary Figure 6 | Median ozone over Africa and Southeast Asia. Median of SHADOZ ozonesonde observations for January and February 2014 (red) and CAM-Chem O_3 sampled on the same days as the ozonesondes (blue) over Nairobi, Kenya (**a**) and Hanoi, Vietnam (**b**). Error bars represent the 25th and 75th percentiles.

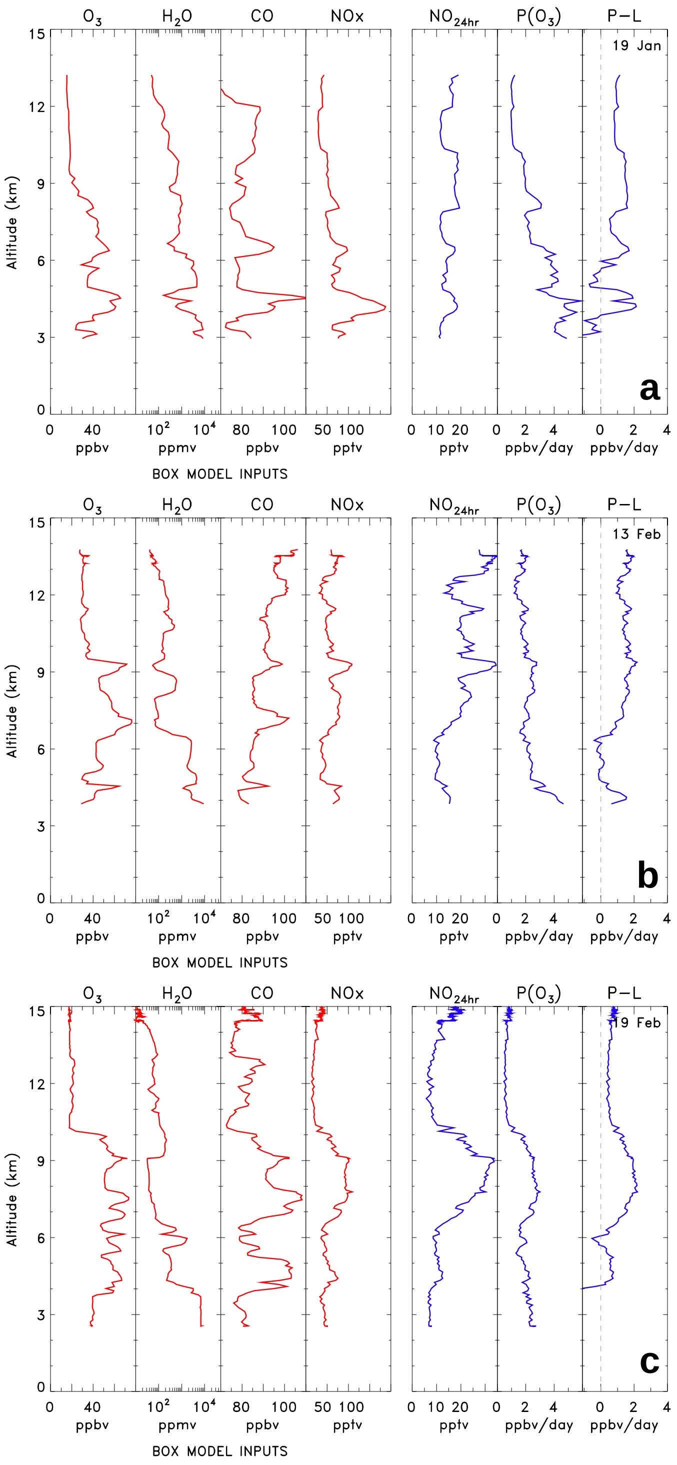

Supplementary Figure 7 | Photochemical ozone production from three CONTRAST profiles. Vertical profiles of observed O_3, H_2O, CO, and NO_x (red) from the CONTRAST flights in Figure 2 used as input for the DSMACC photochemical box model. Model outputs (blue) are 24-hour averaged NO, gross O_3 production ($P(O_3)=k_1[NO][HO_2]+k_2[NO][CH_3O_2]$), and net O_3 production ($P(O_3)^{Net}=P(O_3)-k_3[O_3][OH]-k_4[O_3][HO_2]-k_5[O^1D][H_2O]$). Panels show data from (**a**) 19 Jan 2014 (**b**) 13 Feb 2014 (**c**) 19 Feb 2014.

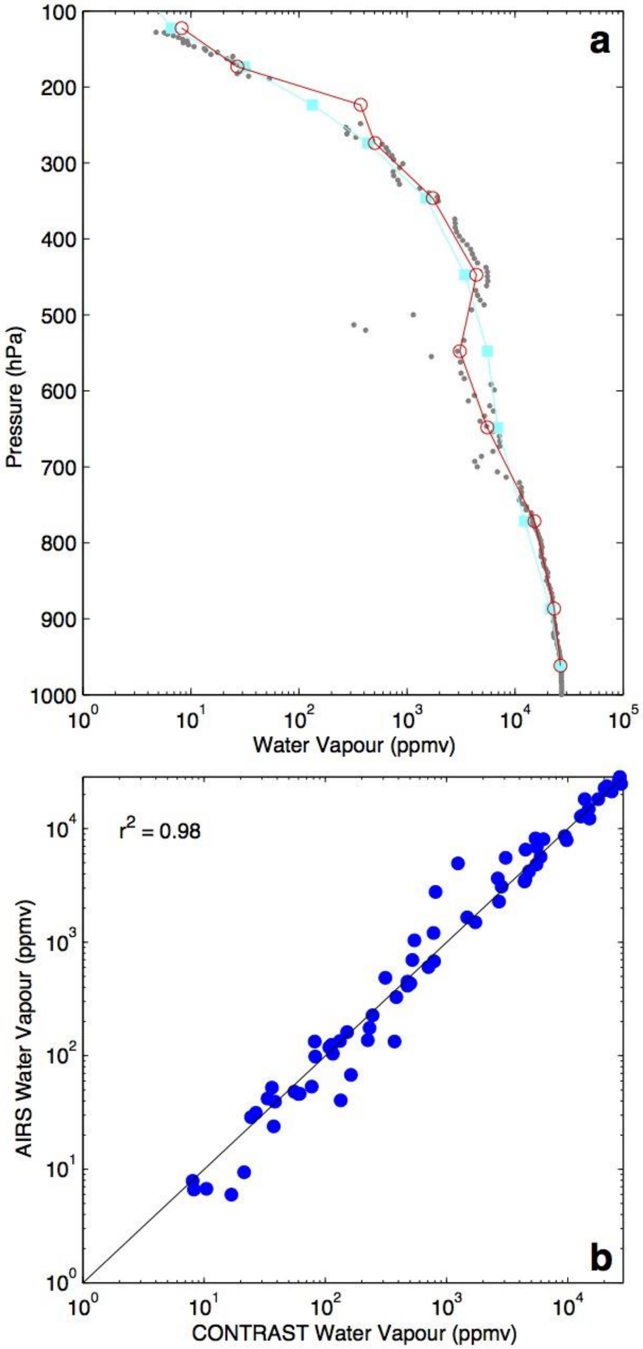

Supplementary Figure 8 | Comparison of AIRS water vapour to *in situ* observations. (**a**) Vertical profile of H_2O mixing ratio from CONTRAST RF11 conducted at 7.1°N and 143°E on 13 Feb 2014. Grey dots are *in situ* observations, red, open circles are *in situ* observations averaged over the AIRS layers, and cyan, closed squares are AIRS observations. AIRS data have been bilinearly interpolated to the profile coordinates. (**b**) *In situ* H_2O from one profile from every CONTRAST flight, averaged to the AIRS layers, regressed against AIRS H_2O interpolated to the respective profile location.

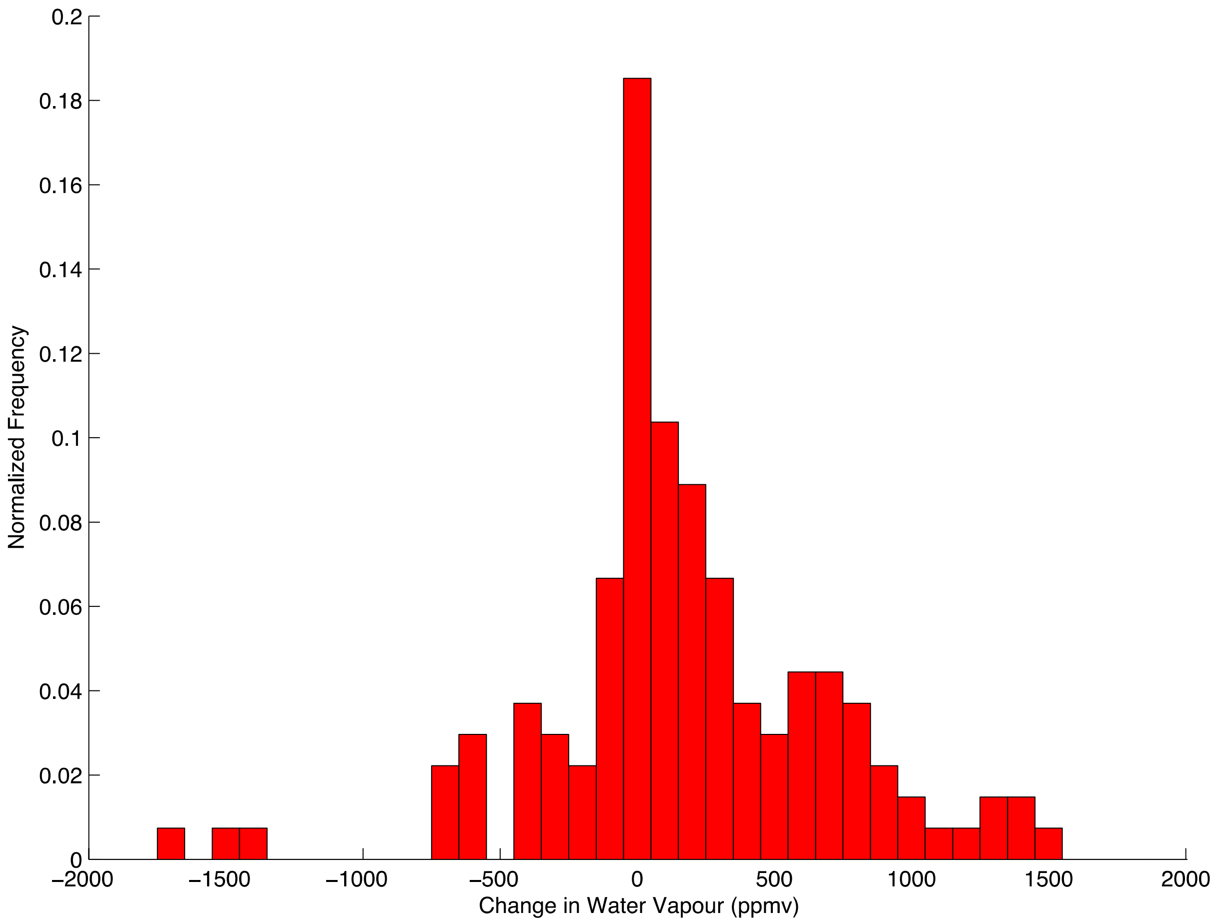

Supplementary Figure 9 | Change in water vapour of air parcels while in transit to the TWP. Normalized distribution of the change in water vapour mixing ratio along the back trajectories for the HOLW structures. The change in water vapour is defined as the difference between the H_2O mixing ratios at the trajectory starting point (*i.e.* the trajectory initialization point along the flight track) and the trajectory end point (*i.e.* 10 days prior to observation or the point of last precipitating convection). Water vapour was calculated from the RH output by the HYSPLIT model, which is based on GDAS meteorology (see methods). Positive values indicate the water vapour mixing ratio has increased during transit to the TWP.
